# Blue and green ammonia production: A techno-economic and life cycle assessment perspective

**DOI:** 10.1016/j.isci.2023.107389

**Published:** 2023-07-14

**Authors:** Patricia Mayer, Adrian Ramirez, Giuseppe Pezzella, Benedikt Winter, S. Mani Sarathy, Jorge Gascon, André Bardow

**Affiliations:** 1 Energy & Process Systems Engineering, Department of Mechanical and Process Engineering, ETH Zurich, 8092 Zurich, Switzerland; 2 King Abdullah University of Science and Technology, KAUST Catalysis Center (KCC), Thuwal 23955, Saudi Arabia; 3 Catalysis Hub, SwissCAT+ East, ETH Zürich, 8093 Zurich, Switzerland; 4 King Abdullah University of Science and Technology, Clean Combustion Research Center (CCRC), Thuwal 23955, Saudi Arabia

**Keywords:** Energy resources, Energy policy, Energy engineering, Energy Modeling

## Abstract

Blue and green ammonia production have been proposed as low-carbon alternatives to emissions-intensive conventional ammonia production. Although much attention has been given to comparing these alternatives, it is still not clear which process has better environmental and economic performance. We present a techno-economic analysis and full life cycle assessment to compare the economics and environmental impacts of blue and green ammonia production. We address the importance of time horizon in climate change impact comparisons by employing the Technology Warming Potential, showing that methane leakage can exacerbate the climate change impacts of blue ammonia in short time horizons. We represent a constrained renewable electricity availability scenario by comparing the climate change impact mitigation efficiency per kWh of renewable electricity. Our work emphasizes the importance of maintaining low natural gas leakage for sustainability of blue ammonia, and the potential for technological advances to further reduce the environmental impacts of photovoltaics-based green ammonia.

## Highlights

• Economic and environmental comparison of blue and green ammonia production
• Consideration of time horizon in climate change impact comparison
• Natural gas leakage exacerbates environmental impacts of blue ammonia
• Environmental impacts of green ammonia are driven by high electricity requirements

## Graphical abstract

**Figure:**
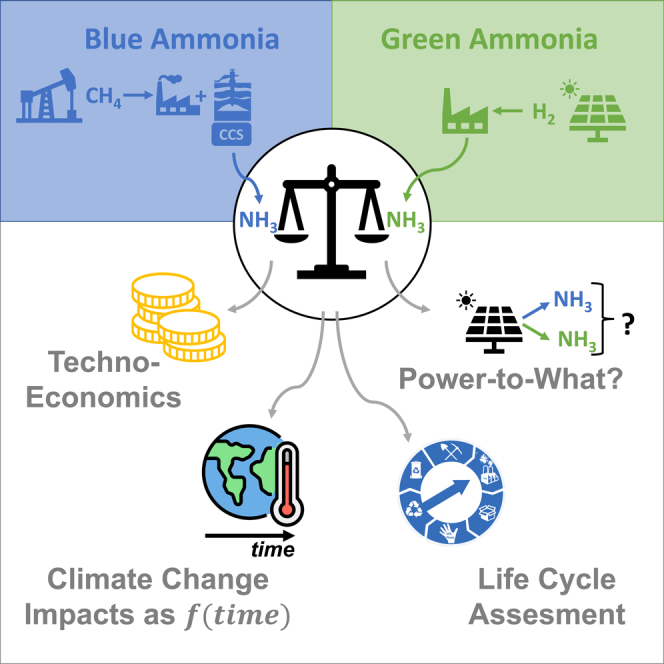

## Introduction

In 1914, Haber and Bosch designed an industrial process based on an iron-based catalyst to produce ammonia from nitrogen and hydrogen mixtures (Equation 1).^1^

Equation 1 $$N_{2}+{3H}_{2}⇌{2NH}_{3}\Delta H^{0}=-10.9\frac{kcal}{mol}\;\Delta G\leq 0\;if\;T\leq 190˚C$$

This simple process saved the world. As a precursor for fertilizer production, ammonia increased food availability and satisfied the world population demand, leading to rapid demographic growth.^2^ Erisman et al.^3^ estimated that the world population would have been 4 billion fewer people without implementing the Haber-Bosch process, making this process vital for humankind.

Nowadays, ammonia has the potential to again play a substantial role in a changing the world toward a low carbon future. Ammonia has a lower heating value of 18.6 MJ/kg, around 40% of the energy density of gasoline, but with a carbonless combustion. Additionally, ammonia has a gravimetric hydrogen content of 17.8%, among the highest of energy carriers.^4^ However, its main advantage is the volumetric energy density of 12.7 MJ/L, three times higher than the volumetric density of compressed hydrogen at 69 MPa and 25°C, and roughly ten times higher than the volumetric density of lithium-based batteries. Therefore, ammonia ranks among the most promising candidates to carry, store, and ship renewable energy around the globe.^5^ Moreover, a full liquid ammonia transport network is already available and used in the fertilizer industry. This infrastructure and existing regulations could be leveraged to guarantee reliable, ready, and safe ammonia distribution as a low-carbon energy vector to be further decomposed back into hydrogen or used directly as fuel.^6^

New technologies such as MILD/flameless combustion will help with ammonia’s penetration as a fuel, starting with furnaces and boilers, where neat ammonia^7^ or ammonia blended with low molecular weight alcohols^8^ have already been shown to work. The use of ammonia extends to co-firing in kilns, decreasing conventional fossil fuel combustion and CO_2_ emissions.^9^ However, ammonia is predicted to above all play a part in the decarbonization of fuels for the marine sector, limiting N_2_O/NO_x_ emissions, without any SO_x_ nor particulate emissions compared to heavy oils.^10^

While ammonia has attractive characteristics as a carbon-free and zero-GWP energy vector, ammonia presents some safety risks due to its corrosiveness and toxicity for humans. Gaseous emissions of nitrogen oxides, ammonia, and sulfur oxides form fine particles that are potentially dangerous for the human respiratory system.^11^ Carbon steel dip-coated with alloys such as ZnAlMg is recommended to prevent ammonia material corrosion, while guidelines limit ammonia exposure between 25 and 35 ppmv to 8 h to reduce risk to human health.^12^

The main differentiator between most present-day ammonia production processes arises from the hydrogen production route that is coupled with the Haber-Bosch process (HB). Currently, more than 96% of the worldwide hydrogen production for ammonia comes from fossil resources: Over 70% of hydrogen for ammonia is generated via steam methane reforming (SMR), using natural gas as a feedstock, and emitting 2.5–2.9 kg CO_2-_eq/kg NH_3_.^13^ Even worse for the climate, coal gasification provides 26% of the hydrogen, mostly in China, emitting 5.2 kg CO_2-_eq/kg NH_3_. Partial oxidation of heavy fuel oil accounts for less than 1% of global production and ammonia synthesis with hydrogen from electrolysis represents only isolated cases. The fossil-based routes are classified as gray ammonia if no CO_2_ capture and storage (CCS) is implemented, or blue ammonia if CCS is applied. The route with hydrogen from electrolysis is defined as green ammonia if renewable electricity is used. Overall, ammonia production consumed 2.4 TWh of energy, 2% of the final global energy consumption, and caused 1% of global greenhouse gas (GHG) emissions in 2020.^6^

The Stated Policies Scenario of the International Energy Agency (IEA) foresees a global population increase of 25% between 2020 and 2050.^14^ A growing population requires higher agricultural and industrial production, expecting to increase ammonia production from 185 Mton in 2020 to 230 Mton by 2050. Additionally, the energy sector is expected to need an additional 125 Mton of ammonia as an energy carrier or as carbonless fuel, increasing ammonia demand from today by 90% till 2050.^14^ The higher demand for ammonia could result in an unprecedented energy consumption and GHG emissions.^15^ These discouraging forecasts demand the search for more sustainable ammonia pathways mandatory to be in line with the recent climate goals.^16^

Several studies assessed the sustainability of hydrogen production,^17^^,^^18^^,^^19^^,^^20^^,^^21^^,^^22^^,^^23^^,^^24^^,^^25^^,^^26^^,^^27^^,^^28^ highlighting important sensitivities. For instance, a recent study from Howarth and Jacobsen^18^ claimed that blue hydrogen emissions are only 9–12% lower than gray hydrogen emissions due to low CO_2_ capture rates and extra energy needed for CO_2_ capture, which requires more natural gas and, therefore, causes more methane leakage. Additional works have been published in response to these claims,^19^^,^^20^ showing that the global warming potential of blue hydrogen is highly sensitive to the natural gas supply chain leakage assumptions, the modeled CO_2_ capture rates, and the global warming potential time horizon. Therefore, these sensitivities are key to such studies and to implementing the processes.

Similarly, the sustainability of ammonia production routes has also been compared.^29^^,^^30^^,^^31^^,^^32^^,^^33^^,^^34^^,^^35^^,^^36^^,^^37^^,^^38^^,^^39^^,^^40^^,^^41^^,^^42^ However, most studies do not consider the integrated SMR-HB process, which has been optimized for decades, but rather evaluate the various hydrogen production processes. Moreover, some studies only rely on theoretical thermodynamic values of blue and green ammonia and hydrogen without a proper process techno-economic analysis (TEA). Most studies also only focus on the climate change impact (CCI) that, while being important, is not the only metric needed to evaluate process sustainability. Studies reporting global warming potential (GWP) also focus on standardized GWP metrics, only considering single points in time. These studies neglect the time-dependency of climate change impacts, which corresponds to the nature of the GHGs emitted (e.g., short-term vs. long-term climate pollutants).^43^

Chisalita et al.^30^ performed a cradle-to-gate life cycle assessment (LCA) of European ammonia production from various hydrogen sources. They located the processes in Germany and identified a scenario with the lowest GWP-based climate change impacts through a sensitivity analysis. The lowest climate change impacts were reported for electrolysis-based hydrogen from renewables, equal to 0.15 ton CO_2_ eq/ton NH_3_. Bicer et al.^31^ evaluated 15 ammonia generation techniques, located the plant for the case studies in the USA, and considered hydrogen and nitrogen generation separately. Besides considering ammonia from SMR, they analyzed coal gasification, partial oxidization of heavy-fuel oil, and green ammonia with hydrogen from electrolysis powered using nuclear, oil, or coal. They found that nuclear-based ammonia production had the lowest climate change impacts (based on GWP500) with 0.48 ton CO_2_ eq/ton NH_3_, while the highest climate change impacts came from ammonia from electrolysis from coal electricity with 13.6 ton CO_2_ eq/ton NH_3_. Liu et al.^38^ compared several technologies for nitrogen and hydrogen production. Pressure swing adsorption (PSA) or cryogenic distillation were used to separate nitrogen from air, while hydrogen derived from four electrolysis technologies was considered: low-temperature electrolysis, high-temperature electrolysis, hydrogen as a by-product of the chlor-alkali process, or as secondary product from steam cracker plants. They evaluated different electricity mix grids and found that the energy source used to power the plant significantly affected the climate change impacts (based on GWP100), with up to 90% deviations in some cases. D’Angelo et al.^42^ performed a planetary boundaries analysis of various low-carbon ammonia production routes while also considering hydrogen production separately. Arora et al.^29^ looked at how raw materials, production processes, and locations affect the costs and emissions of biomass-based ammonia production. They found that the Brazilian bagasse autothermal reforming process had the lowest climate change impacts (based on GWP100) of 0.242 ton CO_2_ eq/ton NH_3_ while the Indian straw CO_2_ reforming plant had the highest with 1.44 ton CO_2_ eq/ton NH_3_, being nevertheless the most economical.

We can observe that, depending on the assumptions made, the climate change impacts of ammonia production greatly vary among the studies (see Table S1 for a detailed overview). The main differences arise from the hydrogen production route selected, the local electricity mix, the natural gas supply chain, the general assumptions of the model, and the GWP time horizon used.

In this work, we present a TEA and full LCA of the integrated blue and green ammonia production processes to compare the environmental impacts and economics of both routes. Our case study is based in Saudi Arabia due to the high potential for both blue and green processes and the current active investment in both. We focus on solar (PV) based electricity due to the case study location while performing a sensitivity on the average solar irradiation yield to generalize the results to other countries. Sensitivities on the natural gas supply chain, CCS supply chain, electricity mix, flue gas capture, and projected technological improvements were also carried out to support our findings. To investigate the importance of time horizon in climate change impact comparisons, we employ the technology warming potential (TWP)^44^ to evaluate the time evolution of the relative cumulative radiative forcing of blue vs. green ammonia. We also consider a constrained renewable electricity availability scenario by comparing the blue vs. green environmental impact mitigation per kWh of electricity, introduced as the power-to-X efficiency by Sternberg et al.^45^ We compare the power-to-X efficiency of the integrated SMR-HB process to the efficiency of a separated SMR plus HB process to highlight the benefits of process integration.

## Results and discussion

### Techno-economic analysis

We perform a TEA of both green and blue ammonia production processes to assess the energy consumption and the impact of the different process parameters on the profitability. The simulation details, process flowsheets, and comparison with advanced blue and green ammonia industrial processes^37^ can be found in the STAR methods section and in the supporting information. The mass balance and compositions of the different streams for both processes are shown in Tables S3 and S4. A simplified block flow diagram of both routes is shown in Figure S2. Both routes produce the ammonia stream in the required purity.

**Figure 1:**
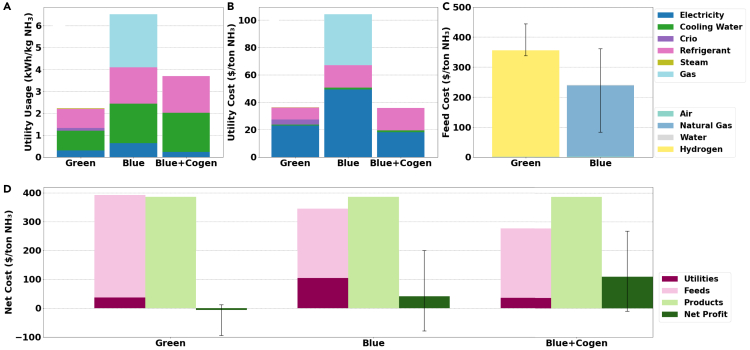
Techno-economic comparison of blue and green ammonia production Summary of the techno-economic analysis for both green and blue processes, with and without cogeneration (Cogen). (A) Utility usage per kg of ammonia. (B) Utility cost per kg of ammonia. (C) Feed cost per kg of ammonia. (D) Net cost per kg of ammonia. Error bars correspond to the following price ranges discussed below in the main text: P_Hydrogen_ = [$1.9/kg, $2.5/kg], P_Natural gas_ = [$2/MMBTU, $9/MMBTU].

The total energy consumption per kg of ammonia of the blue process (6.5 kWh/kg NH_3_) is higher than for the green process (2.2 kWh/kg NH_3_) (Figure 1A). However, it must be noted that here the energy for green hydrogen production is not considered as part of the green process. Hydrogen is rather considered a feed (see Figure S2), with its costs being accounted for accordingly (Figure 1C). It is also important to notice that the blue process contains a purge rich in methane and hydrogen (see PURGE2 in Table S4) and, in the actual industrial process, this stream is not discarded and instead used in a cogeneration plant to provide heat and power to the whole system. The cogeneration drastically reduces the utility consumption of the blue process (3.8 kWh/kg NH_3_), with the heating requirements being wholly obviated. To validate our results, we have compared them with the reported data of the state of the art (see Table S5), showing similar results as the ones presented here. Our values are also similar to the ones obtained with a kinetic model on the NH_3_ synthesis loop instead of an equilibrium-based approach.^46^

Looking in detail at the different contributions of Figure 1A, we can observe that the electricity (for the compressors), water (for general cooling), and refrigerant (for the ammonia separation) are the main contributors to the green process energy requirements. The cryogenic needs for the separation of nitrogen are lower in comparison to the other contributions. When looking at the blue process, we can observe that most of the contribution comes from the ammonia separation refrigerant, cooling water, and gas to heat the reactors, with the electricity for compression being non-dominant. If we now include the cogeneration unit (Cogen), the gas for heating the reactors is provided directly, and the electricity requirements are reduced by circa 40%. However, the energy consumption is almost 70% higher than for the green process (2.2 vs. 3.7 kWh/kg NH_3_) where the energy for providing green hydrogen as feedstock has been neglected.

In Figure 1B, we can observe that despite consuming more total utilities, the corresponding cost of the blue process with the cogeneration is lower than the utility cost of the green process. This finding is due to the fact that the green process uses mostly electricity. Thus, the results confirm that the profitability of the green process will depend strongly on the electricity price (Table S2). Nevertheless, we can observe that the cost of the feed dominates the overall costs of the green process (Figure 1C). The different market prices were estimated as 0.0011 $/kg of air, 0.3031$/kg of CH_4_, 0.0011$/kg of water, 0.3858 $/kg of ammonia, and 2 $/kg of hydrogen for the base scenario. The natural gas price (circa 6$ per MMBTU) is well within the range of the Henry Hub Natural Gas Spot Price, which has traded between $2 and $12 per MMBTU since 1998. The green hydrogen price is slightly more optimistic as, while prices close to 2.5$/kg can be currently achieved in the Middle-East when using alkaline electrolyzers, these prices are not realistic in Europe at least until 2050.^47^ Despite the optimistic hydrogen price, the total overall net costs (*products – feeds – utilities*) are negative for the green process, indicating that, with the considered prices, the process is not profitable (unless a green premium would be added to the price of ammonia). On the other hand, the blue process displays a positive net revenue with and without the cogeneration.

Both routes depend strongly on the feed cost rather than the energy costs (see Figure 1D), with hydrogen and natural gas dominating almost 100% of the feed costs for the green and blue processes, respectively (see Figure 1C). Hence, slight variations in the hydrogen and natural gas market prices will significantly impact the profitability of both processes. To reach breakeven (i.e., (*products = feeds + utilities*), the natural gas price of the blue process with cogeneration could be increased by 50% to 9$ per MMBTU, indicating some margin for the market fluctuations. On the other hand, for the green process, the hydrogen price needs to be reduced by about 24% to 1.9$ per kg to reach breakeven with a 385.8$ per ton price of ammonia. Otherwise, the ammonia needs to be subsided by circa 4% to a selling price of 400$ per ton with a hydrogen price of 2$ per kg, and up to 500$ per ton with the more realistic hydrogen price of 2.5$ per kg. Nevertheless, we must remark that the cost of both feeds will inevitably also depend on the price of electricity, especially the hydrogen from electrolysis for the green process, which is extremely sensitive to the wholesale power price.^47^ We also need to stress that, despite the economic attractiveness of the blue process from our TEA, the economics associated with CCS scale-up to match global ammonia production might not be as straightforward due to its complexity and the many parties involved.^48^

### Life cycle assessment

#### Climate change impacts based on the global warming potential (GWP100)

**Figure 2:**
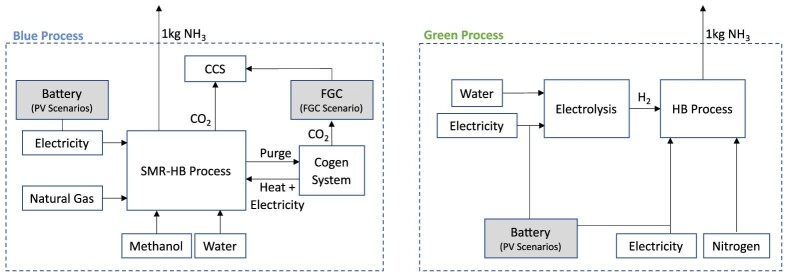
Life Cycle Assessment system boundaries for blue and green processes Life Cycle Assessment scope and system boundaries of blue (left) and green (right) ammonia (*NH*_*3*_) processes. Gray boxes are included only in certain scenarios.

The Global Warming Potential GWP100 metric has become the default metric for calculating and reporting climate change impacts. The United Nations Convention on Climate Change (UNFCCC) requires the use of the GWP100 as a way to equate all emissions to a CO_2_-eq.^49^ The GWP100 lumps all emissions to calculate the cumulative radiative forcing as a CO_2_-equivalent over a 100-year period following a pulse of emissions. However, due to the different lifetimes of different emission types in the atmosphere, the GWP100 does not capture the time dependency of radiative forcing well. To capture these time dependencies, the TWP by Alvarez et al.^44^ is discussed in Section blue vs. green ammonia as a function of time. The system boundaries for the life cycle assessment discussed in the following sections is shown in Figure 2.

**Figure 3:**
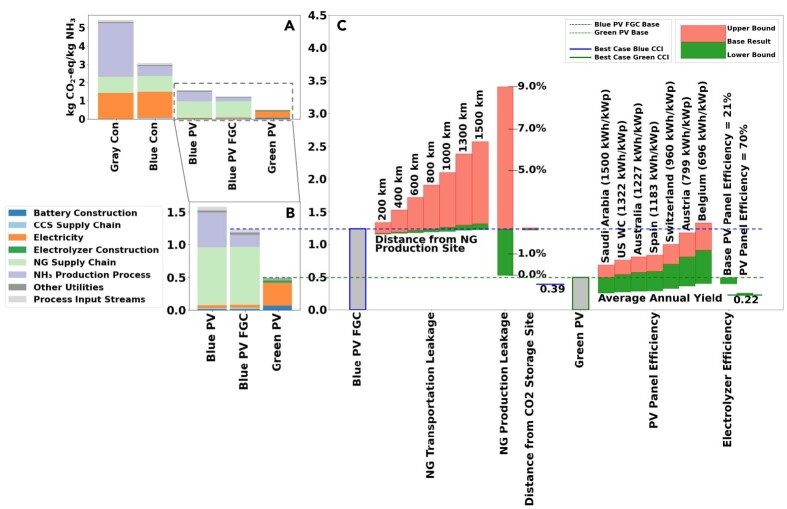
GWP100-based climate change impact comparison of blue and green ammonia production Climate change impact (CCI) comparison and breakdown across different scenarios, including a sensitivity analysis on key supply chain and technological components. (A) CCI comparison across all scenarios, varying the ammonia production process (*Gray, Blue, and Green*) and the electricity mix. *Con* refers to conventional electricity, and *PV* refers to photovoltaic electricity. (B) CCI comparison across photovoltaic electricity scenarios, including a scenario with flue gas CO_2_ capture (*FGC*) for the blue process, (C) Sensitivity analysis on key components. Base parameter values and ranges are given in Table 2. Red/green bars represent increasing/decreasing climate change impact vs. the base parameter value. For the blue process, sensitivity components include leakage rates for natural gas *(NG)* production and transportation, distance from natural gas production site, and distance from CO_2_ storage site. For the green process, sensitivity components include average annual solar irradiation yield, PV panel efficiency, and electrolyzer efficiency.

Figure 3 compares the climate change impacts based on the GWP100 of all the scenarios with base parameter values (see Table 1 for scenarios; Table 2 for the base parameter values, more details in Section scenarios and sensitivities). Using conventional electricity (Figure 3A, Con scenarios), carbon capture and storage (CCS) reduces climate change impacts by 43% in the Blue Con scenario compared to the Gray Con scenario. The conventional process already separates CO_2_ into a concentrated stream at 75bar. Thus, little additional compression and infrastructure are needed to transport and store the CO_2_. This setup renders conventional SMR-HB processes as a particularly promising point source of CO_2_, as also shown by von der Assen et al.^50^

**Table 1:** Life cycle assessment (LCA) scenarios

Electricity scenario	Ammonia production scenario	LCA scenario name
Conventional	Conventional	Gray Con
	Blue	Blue Con
Renewable (PV-based)	Blue	Blue PV
	Blue with Flue Gas CO_2_ Capture	Blue PV FGC
	Green	Green PV

**Table 2:** Base parameters and sensitivity ranges

Production Route	Parameter	Base value (Source)	Sensitivity range	Range justification
Blue	NG Production leakage	2.2% (global average leakage)^51^	0-9%	9.4% from^52^ corresponds to Permian Basin in US as highest value.
				Literature suggests most emissions between 0.5 and 3%^20^
	NG Transportation leakage	0.2% per 1000 km (ecoinvent)	0-3%	Encompasses 0.8% from^18^ and tests extreme cases
	Distance from NG production site	800 km (ecoinvent)	0-1500 km	Maximum length of Saudi Arabia
	Distance from CO_2_ storage site	800 km (consistency with above)	0-1500 km	Maximum length of Saudi Arabia
Green	Electrolyzer Efficiency	60%^53^	60-80%	80% from^54^
	PV Panel Efficiency	21% (average efficiency of silicon HIT PV modules)^55^	13.34–70%	13.34% corresponds to efficiency in ecoinvent.
				Theoretical PV efficiencies can go up to 70%^56^
	Average Annual Yield	1500 kWh/kWp (ecoinvent^57^)	696-1500 kWh/kWp	Ecoinvent^57^ values for different countries with Belgium representing the lower end

Under the photovoltaic (PV) electricity scenarios where PVs provide steady-state electricity with help of battery storage (Figure 3B, PV scenarios), the green process has 69% lower climate change impacts than the blue process. The blue process’ impacts are dominated by the natural gas leakage and the flue gas emissions from the cogeneration system. If flue gas CO_2_ capture (FGC) is introduced to the blue process, the blue process emissions are reduced by 22% (Blue PV FGC scenario compared to the Blue PV scenario). Additional emissions come from the CCS supply chain because the flue gas system is operating at 1 bar and thus requires more electricity for CO_2_ compression. Under the base parameter conditions with PV electricity, the climate change impacts of the green process are 60% lower than those of the blue process with flue gas capture (0.5 kg CO_2_-eq vs. 1.2 kg CO_2_-eq).

Figure 3C shows a sensitivity analysis of key supply chain and technological components (see Table 2 for the base parameter values and sensitivity ranges). The base results for the sensitivity analysis correspond to the Blue PV FGC and the Green PV scenarios from Figure 3B. The climate change impacts of the blue process depend strongly on the natural gas supply chain leakage: if natural gas production leakage is decreased from the base parameter value of 2.2%, representing the global average,^51^ by a factor of 10, climate change impacts of the blue process decrease by 50%. The low leakage rates have been reported for Algeria^57^ and by companies,^58^ and thus seem technically feasible. However, under the high-end natural gas leakage rate of 9% observed in US gas fields,^52^ the climate change impacts of the blue process are nearly tripled. Therefore, it is crucial to closely monitor and limit natural gas leakage to ensure low climate change impacts for the blue process.^51^ The sensitivity analysis also shows that the distance from the CO_2_ storage site does not have a large effect on the climate change impacts relative to the other sensitivities performed.

The climate change impacts of the green process with photovoltaic (PV) electricity are driven by the electrolysis electricity requirement, the impacts of which are driven by the PV supply chain. This finding is in line with Palmer et al.,^27^ who also show that the GHG emissions of electrolysis via solar PV are dominated by the PV modules. At the base parameter value for PV panel efficiency of 21%, representative of the average efficiency of silicon HIT PV modules,^55^ the climate change impacts of the green process are lower than those of the blue process under base conditions even for countries with lower average irradiation, such as Belgium (696 kWh/kWp). However, lower PV efficiencies around 13% can yield higher climate change impacts for the green process than for the blue process under base conditions (Figure 3C). This finding emphasizes the importance of technological improvements for reducing the climate change impacts of the green process.

The electrolyzer efficiency sensitivity shows that with the high-end electrolyzer efficiency value of 80% at the base PV panel efficiency of 21%, the green process impacts would decrease by 19%. However, the electrolyzer and PV efficiencies are likely to improve simultaneously. The best-case climate change impacts of the green process at an electrolyzer efficiency of 80% and PV efficiency of 70% would be 0.22 kg CO_2_-eq, 44% lower than the best-case blue process climate change impacts of 0.39 kg CO_2_-eq with no natural gas leakage, no CO_2_ leakage from CCS, and 70% PV panel efficiency. For reference, the climate change impacts of the green process would reach an optimistic value of 0.23 kg CO_2_-eq using current on-shore wind electricity under base parameter values, without considering the battery system. Details of this benchmark scenario can be found in the supplemental information.

#### Blue vs. green ammonia as a function of time

While the GWP100 has become the *de facto* standard climate change metric, its static view on global warming potential has its limitations since different greenhouse gases (GHGs) have different effects on the climate depending on the time horizon considered.^59^ Methane (CH_4_), a short-lived GHG, has a stronger immediate radiative forcing than CO_2_ but decays much faster. On a mass basis, methane has approximately 120 times more radiative forcing than CO_2_ directly following its emission but only lasts about 12 years in the atmosphere before decaying.^60^ Therefore, the cumulative radiative forcing from a methane molecule is larger in shorter time horizons than in longer time horizons. The time horizon considered plays a role in the GWP calculation, which compares the cumulative radiative forcing to that of CO_2_.

Different metrics have been developed to address the limitation mentioned above. The GWP20, for example, calculates the global warming potential over 20 years rather than 100, thus placing a bigger weight on methane emissions. Recent literature has emphasized the importance of separating long and short-lived GHGs in climate change calculations. The choice of metric and method applied to calculate the effect on climate from different GHGs is a topic under development.^49^^,^^59^^,^^61^^,^^62^

**Figure 4:**
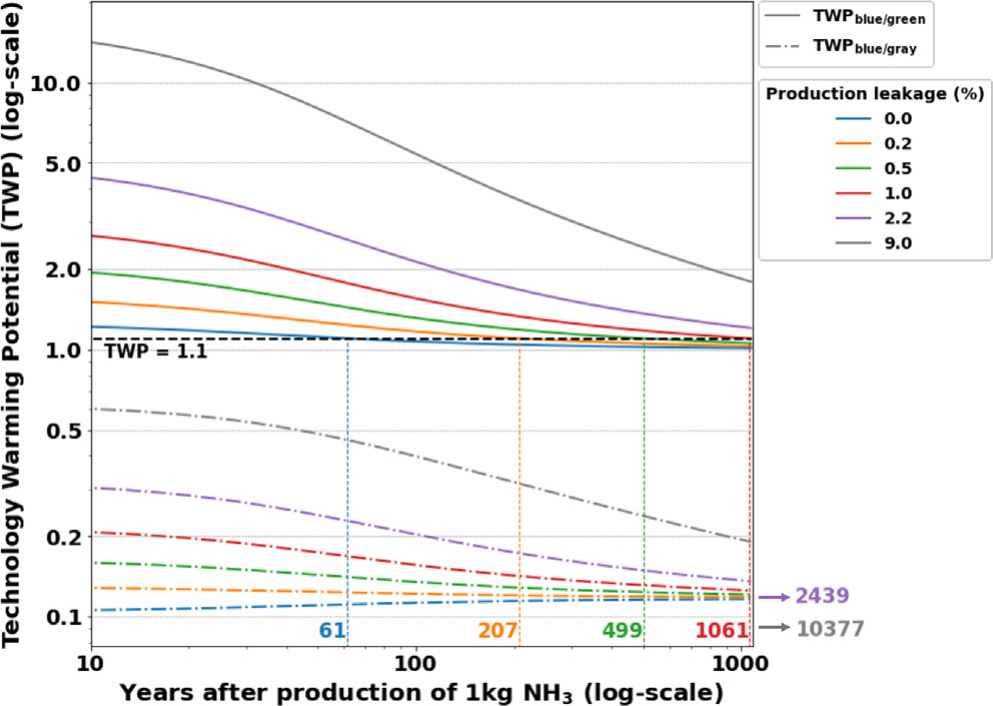
Technology Warming Potential (TWP) of blue ammonia production relative to green and to conventional ammonia production Technology Warming Potential of blue vs. green ammonia production (TWP_blue/green_) as solid lines and of blue vs. gray ammonia production (TWP_blue/gray_) as dashed lines with varying natural gas production leakage rates. Base parameter values were used along with PV electricity except for natural gas production leakage, which is varied. The colored numbers indicate the years when the cumulative radiative warming of the blue process reaches 110% that of the green process for the various natural gas production leakage rates (TWP_blue/green_ = 1.1).

To study the relative cumulative radiative forcing of two technologies with differing CO_2_ and methane emissions as a function of time, Alvarez et al.^44^ introduced the TWP. We apply the TWP to blue vs. green ammonia (TWP_blue/green_) and to blue vs. gray ammonia (TWP_blue/gray_) to understand the time evolution of the relative cumulative radiative forcing, *t* years following the production of 1 kg ammonia (Figure 4). PV-based electricity and the base parameter values are used except for natural gas production leakage rates, which we vary.

The TWP_blue/green_ is largest in the early years following production as a result of methane emissions from blue ammonia, which increase its radiative forcing in short time horizons. The TWP_blue/green_ decreases as the methane decomposes and stabilizes around a value of 1 for all leakage rates. The stabilized value of 1 indicates that, under base parameter conditions, the cumulative radiative forcing of blue ammonia is at-best equal to that of green ammonia even over long-time horizons because blue and green ammonia emit similar amount of CO_2_. However, the time to reach a TWP_blue/green_ of 1 varies drastically depending on the leakage rate: With no natural gas production leakage, it takes 61 years for the blue process cumulative radiative forcing to come within 10% of the green process cumulative radiative forcing. However, with a natural gas leakage rate of 9%, it takes over 10,000 years for the 10% threshold to be met (Figure 4). Therefore, while green ammonia under base conditions outperforms blue ammonia with respect to climate change impacts regardless of natural gas leakage, reducing natural gas leakage can drastically improve the relative climate change impacts of blue ammonia, particularly in short time horizons.

Additionally, the TWP_blue/gray_, comparing blue to gray ammonia, is always less than 1 and stabilizes around a value of 0.1, indicative of the ratio of CO_2_ emissions between the two processes. Since gray ammonia production also requires the separation of CO_2_, the main difference in emissions between gray and blue ammonia comes from the release vs. the capture of the concentrated CO_2_ stream (Table S4.1, Stream CO_2_). The emissions associated with the CCS supply chain and with flue gas CO2 capture for the blue process are not as significant because they are driven by PV electricity. With high natural gas leakage, the TWP_blue/gray_ starts closer to 1 in short time horizons because the radiative forcing of both gray and blue ammonia is driven by the fugitive methane emissions of the natural gas supply chain, which are the same for both processes. With low natural gas leakage, the TWP_blue/gray_ starts around the stabilization value of 0.1 even in short time-horizons because the radiative forcing of both gray and blue ammonia is driven by the CO_2_ emissions.

The TWP analysis shows that although the climate change impacts of the blue process under base conditions are at-best equivalent to the climate change impacts of the green process even over long time horizons, natural gas leakage has a large effect on the climate change impacts of the blue process relative to both green and gray ammonia in short time horizons. If natural gas leakage can be kept low, not only is the blue process most competitive with the green process in short time horizons, but the blue process also reduces the short-term climate change impacts the most drastically relative to conventional production.

#### Environmental impacts beyond climate change

**Figure 5:**
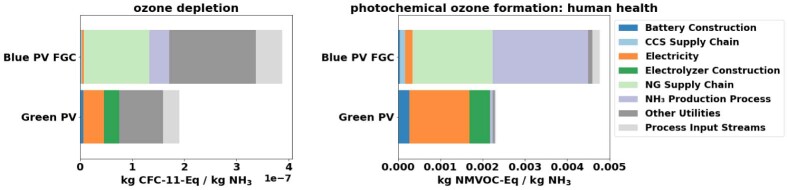
Comparison between blue and green ammonia production of environmental impact categories sensitive to the modeling inputs Blue and green comparison of impact categories sensitive to modeling inputs. These impact categories include Ozone Depletion *(OD)* and Photochemical Ozone Formation *(POF).* The sensitivity analysis results are shown in Figure S4.

**Figure 6:**
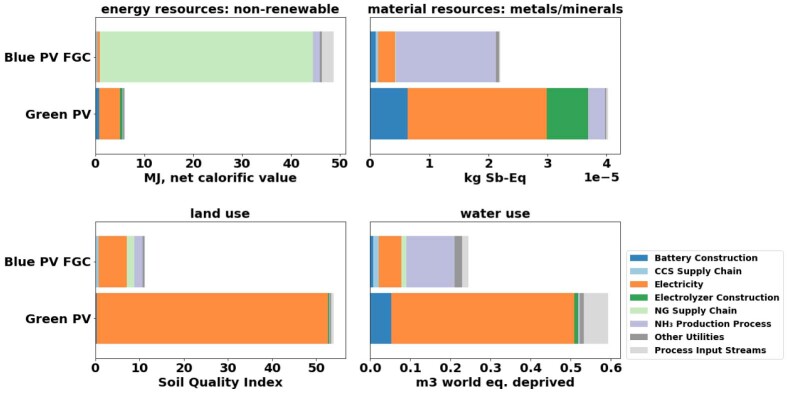
Comparison between blue and green ammonia production of environmental impact categories focusing on resource utilization Blue and green comparison of impact categories highlighting the differences in resource utilization. These impact categories include non-renewable energy use, metals/minerals material use, land use, and water use.

A comparative LCA for all the Environmental Footprint (EF)^63^ 3.0 impact categories in the ecoinvent database version 3.8^57^ was carried out. The database considers the 16 EF environmental impact categories ranging from climate change to human toxicity and thus allows for a holistic assessment. The 15 impact categories besides climate change impact, which is discussed in Section climate change impacts based on the global warming potential (GWP100), were categorized as follows (Table 3): (1) impact categories sensitive to the modeling inputs (Figure 5), (2) impact categories highlighting the differences in resource utilization (Figure 6), and (3) remaining impact categories (Figure S3). All impact categories are sensitive to the average annual irradiation yield and PV panel efficiency due to the large environmental impacts associated with PV supply chains and infrastructure. Due to a lack of trade-offs, these sensitivities are not elaborated below.

**Table 3:** Categorization of environmental impact categories included in LCA

Impact categories sensitive to the modeling inputs	Ozone depletion
	Photochemical ozone formation: human health
Impact categories highlighting the differences in resource utilization	Energy Resources: Non-Renewable
	Material resources: Metals/Minerals
	Land Use
	Water Use
Remaining impact categories	Particulate Matter Formation
	Acidification
	Ecotoxicity: Freshwater
	Eutrophication: Freshwater
	Eutrophication: Marine
	Eutrophication: Terrestrial
	Human Toxicity: Non-Carcinogenic
	Human Toxicity: Carcinogenic
	Ionizing Radiation: Human Health

Figure 5 shows the results of the impact categories sensitive to the modeling inputs, shown in Table 2 and explained in Section scenarios and sensitivities. Under base conditions, both Ozone Depletion (OD) and Photochemical Ozone Formation (POF) have higher impacts for blue ammonia than for green ammonia. Both OD and POF are sensitive to the natural gas supply chain leakage rates (Figure S4).

OD for blue ammonia is mainly driven by the natural gas supply chain due to Halon 1211 leakage, while for green ammonia OD is mainly driven by the PV and electrolysis infrastructures due to carbon tetrachloride (R-10) and CFC-12 leakage. Therefore, OD is sensitive to the natural gas transportation distance due to the leakage of Halon 1211, a component used in fire extinguishers. Due to its ozone-depleting properties, the production of Halon 1211 was banned in most countries as a result of the Montreal Protocol in 1994. Although existing Halon can still be used, new fire extinguishing technologies have emerged, and therefore this sensitivity may not be reflective of modern-day natural gas supply chains.

POF from blue ammonia is mainly driven by methanol leakage, which is used for the CO_2_ capture. POF from green ammonia is mainly driven by the PV and electrolysis infrastructures due to nitrogen oxides, and non-methane volatile organic compounds (NMVOCs) emitted throughout those supply chains. Since nitrogen oxides and NMVOCs are present as emissions of the natural gas supply chain, the POF is sensitive to the natural gas leakage rates.

Figure 6 shows the results of the impact categories indicative of differing resource utilization. Blue ammonia has a higher impact only for non-renewable energy consumption due to its fossil basis. For all others, green ammonia has a higher impact than blue ammonia. This finding is mainly due to the large electricity requirements of green ammonia and the corresponding large resource requirements of PV supply chains. Figure S3 shows the remaining impact categories, mainly affected by emissions from infrastructure supply chains. Except for Carcinogenic Human Toxicity, which shows similar impacts for both blue and green ammonia, the impact categories all follow the same trend, with green ammonia having a higher impact than blue ammonia. For blue ammonia, these impacts come from the chemical facility construction, whereas for green ammonia, they come mainly from the PV and/or electrolyzer infrastructure.

The full LCA shows that blue ammonia performs worse than green ammonia in OD, POF, and non-renewable energy consumption. Both processes have similar impacts regarding carcinogenic human toxicity. Besides these four impact categories, green ammonia has higher impacts than blue ammonia in 11 out of the 15 impact categories besides climate change impacts. These results are primarily due to the large impacts associated with PV and electrolyzers. While these supply chains are expected to improve, the LCA results indicate that blue ammonia at present-day can provide a more sustainable ammonia production option relative to green ammonia from solar PV.

#### Power-to-what?

Until now, 1 kg of ammonia was considered as the functional unit. The renewable electricity scenarios assume an unlimited renewable electricity availability from PV, which is not reflective of present-day renewables availability. A scenario with limited renewable electricity availability, therefore, compares the blue and green climate change impact mitigation per unit of renewable electricity to see which process can most efficiently utilize a marginal unit of renewable electricity to reduce global warming.

We define a Power-to-X efficiency as the difference in climate change impacts between a low-carbon production process and a conventional production process, per kWh of renewable electricity. To compare the Power-to-X efficiencies of blue and green ammonia production, we first calculate the respective quantities of blue and green ammonia, in kg, that could be produced per kWh of renewable electricity. The corresponding climate change impacts for the calculated quantities are then compared to the climate change impacts from producing the same quantity via conventional ammonia production. The resulting difference represents the Power-to-X efficiency for climate change mitigation, thus adapting the methodology of Sternberg et al. for energy storage systems to ammonia production.^45^

**Figure 7:**
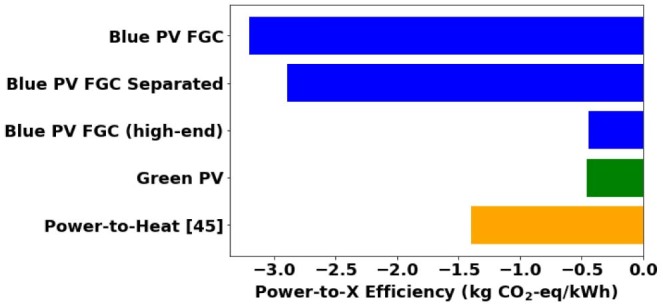
Power-to-X efficiency comparison Comparison of climate change impact reduction vs. conventional production in kg CO_2_-eq per kWh of renewable electricity. The *Blue PV FGC* scenario refers to blue ammonia production with renewable electricity and flue gas CO_2_ capture under base parameter conditions. The *Blue PV FGC Separated* scenario refers to a decoupled blue ammonia process considering hydrogen from SMR followed by ammonia synthesis via the HB synthesis loop. The *high-end parameters* scenario considers the integrated *Blue PV FGC* scenario with the high-end extreme parameter values for distance from the natural gas production site, natural gas production leakage rate, and natural gas transportation leakage rate. The *Green PV* scenario refers to green ammonia production with renewable electricity under base parameter conditions. *Power-to-Heat* refers to the best performer in Sternberg et al.^45^

Figure 7 shows that, per kWh of renewable electricity, the blue process reduces climate change impacts about 7 times more than the green process when compared to producing 1kWh-worth of ammonia with the conventional process under base parameter conditions. The blue process is better than the green process due to the lower electricity requirement per kg ammonia: 1kWh of electricity can produce 0.77 kg of blue ammonia compared to 0.09 kg of green ammonia. Thus, the blue process can substitute more conventional ammonia production, leading to larger climate impact reductions per unit of electricity. Even when comparing the climate change impact mitigation of blue ammonia vs. the best performer in Sternberg et al.^45^ (Power-to-Heat via heat pumps), the blue process performs about two times better, reducing 3.2 kg CO_2_-eq/kWh compared to 1.4 kg CO_2_-eq/kWh. The high efficiency of the blue process arises because conventional ammonia production already separates CO_2_ into a concentrated stream at 75 bar, thus enabling carbon capture and storage with little additional electricity.

Notably, the power-to-X efficiency of the green process is similar to the efficiency of the blue process for a worst-case scenario considering the high-end parameter values (Table 2) for distance from the natural gas production site, natural gas production leakage rate, and natural gas transportation leakage rate. This finding highlights that while green ammonia can better mitigate climate change impacts than blue ammonia on a per kg ammonia basis, green ammonia may pose challenges regarding renewable electricity requirements. The largest green hydrogen parks currently in operation produce around 65 tons H_2_/day,^64^ whereas production volumes of 540 tons H_2_/day would be needed to reach ammonia production capacities of 3000 tons NH_3_/day, in line with blue ammonia production. In the near term, it is not expected that renewable electricity will be abundantly available. Therefore, the blue process might offer a good immediate solution for mitigating the environmental impacts of ammonia production under limited renewable electricity availability, as long as natural gas supply chain leakage rates are monitored and maintained low.

To understand the benefits of SMR-HB process integration, we compare the power-to-X efficiency of the integrated SMR-HB blue ammonia process to the efficiency of a separated process considering hydrogen from SMR followed by ammonia synthesis via the HB synthesis loop. The details of the separated SMR plus HB process representation along with an elaboration of the process integration benefits are provided in the supporting information in Section benefits of process integration. The integrated blue ammonia process mitigates 0.3 kg CO2-eq/kWh more than the separated SMR plus HB process. These results indicate that comparing blue and green ammonia is not equivalent to decoupling and comparing the hydrogen production pathways and that blue ammonia provides a more efficient use of limited renewable electricity for climate-change mitigation than blue hydrogen relative to their conventional alternatives.

### Conclusions

The economic and environmental performance of blue and green ammonia production are compared for a wide range of influencing factors such as natural gas leakage rates and solar panel efficiency. Our results indicate that blue ammonia can be a suitable present-day sustainable ammonia production option if natural gas leakage rates are maintained low. Particularly under present-day conditions, which include constrained renewable electricity availability and high hydrogen prices, blue ammonia can provide advantages over green ammonia as a replacement for conventional ammonia production. Our economic analysis shows that at current ammonia prices, green ammonia would not be profitable even when considering a present-day optimistic hydrogen price.

However, our GWP100-based climate change impact comparison shows that blue ammonia with flue gas CO_2_ capture can only be competitive with PV-based green ammonia when considering natural gas production leakage rates lower than 0.2%. In this case, the climate impacts are similar even in locations with high solar irradiation such as Saudi Arabia. Importantly, the climate change impacts of blue ammonia double at the global average leakage rate of 2.2%,^51^ and increase 7-fold at a high-end leakage rate of 9%, which is currently being observed in some parts of the world.^52^ The importance of keeping natural gas leakage rates low is reiterated when considering the time dependence of climate change impacts. By employing the TWP,^44^ we find that while the cumulative radiative forcing of blue ammonia is at-best equal to the cumulative radiative forcing of green ammonia, reducing natural gas leakage drastically improves the cumulative radiative forcing of blue ammonia, particularly in short time horizons. Beyond climate change, we find that 11 of the 15 impact categories of the product environmental footprints are lower for blue ammonia than for green ammonia.

Considering the current limited availability of renewable electricity, we introduce and compare the power-to-X efficiency of blue and green ammonia. The power-to-X efficiency is defined as the difference in climate change impacts between a low-carbon production process and a conventional production process, per kWh of renewable electricity. We find that the power-to-X efficiency of blue ammonia is seven times that of green ammonia due to green ammonia’s high electricity requirements. While green ammonia can better mitigate climate change impacts on a per kg ammonia basis, blue ammonia might offer an immediate solution for mitigating the environmental impacts of ammonia production under limited renewable electricity availability, as long as natural gas supply chain leakage rates are monitored and maintained low.

The impacts of the green process are mainly driven by the large resource requirements and present-day supply chains of photovoltaics and electrolyzers. Thus, sustainability of green ammonia production can drastically improve with technological and supply chain improvements of the above-mentioned technologies. This finding is reemphasized with the sensitivity analysis of the climate change impact results: The climate change impacts of the best-case green process with an electrolyzer efficiency of 80% and PV panel efficiency of 70% would have 44% lower climate change impacts than the best-case blue process with no natural gas leakage, no CO_2_ leakage from CCS, and 70% PV panel efficiency.

Overall, our study indicates that if natural gas leakage rates can be kept low, blue ammonia production can be a suitable present-day replacement for conventional ammonia production. Additionally, alternative advanced blue ammonia production concepts^37^ could improve the energy efficiency (Table S6), reducing the process energy requirements. Higher efficiencies together with low natural gas leakage could make blue ammonia a promising present-day sustainable replacement for conventional ammonia production while the technologies for green ammonia further develop and the supply chains improve.

### Limitations of the study

While our study aims to provide as comprehensive a comparison as possible between blue and green ammonia production, limitations are still present. One major limitation lies in the process models which do not explicitly consider the flue gas capture for the blue process or electrolysis for the green process. As a result, the techno-economic analysis (TEA) does not resolve the energy and material requirements associated with both of these components. In the TEA, green hydrogen is assumed to be purchased from an external supplier and the flue gas capture is not considered. As a consequence, the LCA regarding these components relies on literature data rather than more detailed process models. Additionally, our process models are not optimized for energy efficiency, which could be improved by employing more advanced production concepts such as those in Arnaiz del Pozo et al.^37^ As a result, the TEA and LCA results of both the green and blue process may be overestimated.

Another limitation lies in the scope for green ammonia, which only considers a detailed evaluation with PV electricity. While a wind electricity benchmark was evaluated and included in the text, further evaluation of wind electricity-based green ammonia is needed for a more complete comparison.

Finally, our study does not consider dynamic electrolyzer operation but rather considers a battery system sized for steady-state electrolyzer operation. Our approach overestimates the size of the battery system and thus the study could be improved by taking a more detailed look into the mode of operation of the electrolyzer and alternative storage systems for hydrogen.

## STAR★Methods

### Key resources table

**Table:** 

REAGENT or RESOURCE	SOURCE	IDENTIFIER
**Deposited data**		

Background data for life cycle assessment	Ecoinvent Database version 3.8^57^	https://ecoinvent.org/the-ecoinvent-database/login/
Inventory for CCS supply chain	Shu et al.^65^	https://doi.org/10.1016/j.rser.2023.113246
Inventory for electrolyzer construction	Bareiβ et al.^53^	https://doi.org/10.1016/j.apenergy.2019.01.001
Inventory for battery construction	Ellingsen et al.^66^	https://doi.org/10.1111/jiec.12072
Method for Life Cycle Impact Assessment	Environmental Footprint Methodology^63^	https://environment.ec.europa.eu/publications/recommendation-use-environmental-footprint-methods_en
Data tables for LCA of blue and green ammonia production	This paper	N/A

**Software and algorithms**		

Aspen Plus V11	aspentech	https://www.aspentech.com/en/products/engineering/aspen-plus
Brightway2 2.3	Brightway Software Framework	https://documentation.brightway.dev/en/latest/index.html
Activity Browser 2.6.7	Steubing et al.^67^	https://doi.org/10.1016/j.simpa.2019.100012 https://github.com/LCA-ActivityBrowser/activity-browser
Python 3.8.10	Python Software Foundation	https://www.python.org/downloads/release/python-3810/

### Resource availability

#### Lead contact

Further information and requests for resources should be directed to and will be fulfilled by the lead contact, André Bardow (abardow@ethz.ch).

#### Materials availability

No materials were used in this study.

### Method details

#### Goal and scope definition

We have performed a TEA and a comparative cradle-to-gate LCA of green and blue ammonia production, focusing on the ammonia production routes rather than on ammonia use or disposal. Due to the comparative nature of the LCA, cradle-to-gate system boundaries are sufficient as later life cycle stage can be assumed to be the same and thus cancel in a comparative assessment.^50^ The functional unit is 1 kg ammonia. Process simulations of both the green and blue ammonia processes were carried out with steady-state simulation models developed in Aspen Plus with a target plant size of 1500 tons NH_3_/day (further details in the supporting information). The process flowsheets of the two ammonia production routes are shown in Figures S5 and S6. The LCA system boundaries are shown in Figure 2.

The case study is based in Saudi Arabia due to the high potential for both blue and green processes and the current active investment in both. The impact assessment method is EF 3.0.^63^ The background system is modeled using the ecoinvent database version 3.8, Allocation, Cutoff by classification.^57^

Two electricity scenarios are included: a fossil-based electricity mix and a renewables-based electricity mix. Taking Saudi Arabia as the case study for this paper, solar (PV) is chosen as the renewable technology. However, a sensitivity is carried out on the average solar yield to generalize the results to other countries.

The renewable electricity scenarios include battery systems. The battery systems are sized for steady-state ammonia production and thus represent conservative estimates. In practice, electrolyzer production can fluctuate to better-match renewables availability and hydrogen can be stored instead of electricity. Thus, battery systems can be smaller than what is included in our analysis. Details of the electricity and the battery system modeling are included in the supplemental information.

#### Green process modeling

The green process consists of three main segments: hydrogen production, air separation, and ammonia synthesis (see Figure S5). The table below summarizes the inputs required for the green process per kg ammonia. Inventory details can be found in the supplemental information.

##### Hydrogen production

Electrolysis is modeled based on Bareiβ et al.,^53^ who provide detailed inventories for the construction of a PEM electrolyzer. The open literature suggests that it is still not clear whether alkaline or PEM electrolyzers will dominate the market for hydrogen electrolysis. However, although alkaline electrolyzers are currently more developed and have been more widely implemented, PEM electrolyzers are often regarded as promising due to their higher efficiencies, better dynamic operation, and higher hydrogen purity.^13^^,^^27^^,^^53^ An electrolyzer efficiency of 60% was used as the base efficiency value to produce hydrogen at 30bar. This value is consistent with Palmer et al.^27^ who also use 55kWh/kg hydrogen even though they model alkaline electrolysis rather than PEM electrolysis.

##### Air separation

Inventories for nitrogen production are adapted from D’Angelo et al.^42^ After purification, nitrogen is compressed to 200 bar with a multistage compressor. Hydrogen is also compressed and mixed with nitrogen.

##### Ammonia Synthesis (HB synthesis loop)

The pressurized mixed feed is first mixed with the reactor recycle and then sent to the ammonia synthesis reactor that follows the Haber-Bosch process ($N_{2}+{3H}_{2}↔{2NH}_{3})$. The reactor operates at 550°C and 200 bar. The outlet stream is sent to a heat exchanger and then to a flash separator to recover the liquefied ammonia. The gaseous stream with unreacted nitrogen and hydrogen is sent back to the reactor to increase the yield.

**Table:** Green process input summary

Section	Technosphere Flow	Quantity (per kg NH3)
Feeds	Hydrogen	0.18 kg
	Nitrogen	0.82 kg
Utilities	Electricity	0.3 kWh
	Refrigeration Electricity (without ASU)	0.44 kWh
	Cooling Water	155.14 kg
Infrastructure	Facility	9.15e-11 units
	Battery (PV scenario)	9.97e-5 kWh capacity

#### Blue process modeling

The blue process (Figure S6) consists of three sections: natural gas reforming, CO_2_ capture, and ammonia synthesis. The table below summarizes all the inputs required for the blue process per kg ammonia. Inventory details can be found in the SI.

##### The natural gas reforming stage

The natural gas reforming stage consists of 3 processes that, after the CO_2_ capture, yield a N_2_ and H_2_ mixture with the perfect stoichiometry (1:3) for the Haber-Bosch NH_3_ reactor: In the first step, the natural gas is pressurized to 35 bar and then mixed with steam before entering the first reformer. The natural gas is transformed into a mixture of CO and H_2_ (${CH}_{4}+H_{2}O↔3H_{2}+CO)$. CO_2_ is also produced as byproduct ($CO+H_{2}O↔{CO}_{2}+H_{2})$. In the second step, a secondary reforming with oxygen takes place.^68^ Here, the unreacted natural gas of the fist reformer is mixed with an air stream and partially oxidized to produce more CO and H_2_ ($2{CH}_{4}+O_{2}↔2CO+{4H}_{2})$. CO_2_ is here also produced as byproduct (${CH}_{4}+{2O}_{2}↔{CO}_{2}+{2H}_{2}O$). The oxygen of the air stream is fully consumed and only a mixture of N_2_, H_2_, H_2_O, CO, CO_2_ and unreacted CH_4_ is left after this stage. Following, this mixture is sent to the 3^rd^ reactor, a Water Gas Shift reactor that transform the CO into CO_2_ and additional H_2_ ($CO+H_{2}O↔{CO}_{2}+H_{2})\text{.}$ A set of heat exchangers is strategically placed between each reactor to minimize the heat consumption. Lastly, the water is condensed and removed in a flash separator before going to the CO_2_ capture stage.

##### TheCO_2_ capture stage

The water-free steam is first recompressed to 75 bar, then enters the CO_2_ Absorber-Stripper capture cycle. The absorption is based on methanol as solvent, similarly to the Rectisol process developed by Linde. After the CO_2_ is captured in the absorber, the methanol is regenerated in the stripper and further cooled down in a heat exchanger before reentering the absorber again. The capture yield is close to 95%. The purified CO_2_ stream from the capture cycle is sent to transport and storage. The CO_2_-free H_2_ and N_2_ rich stream is sent to the ammonia synthesis stage.

##### Ammonia synthesis

This stage is very similar to the green process although some modifications are needed: First, the remaining traces of CO_2_ are removed in a methanizer unit were some H_2_ is consumed to generate CH_4_ $\left({CO}_{2}+{4H}_{2}↔{CH}_{4}+{2H}_{2}O\right)$. Secondly, a purge is used to avoid CH_4_ accumulation in the reaction loop. The NH_3_ synthesis reactor operates at the same conditions as in the green process, 550°C and 200 bar. The outlet stream is also sent to a heat exchanger and then to a series of flash separators to recover the liquefied NH_3_.

The blue process produces a purge stream rich in methane. This stream is used to generate 125% of the heat and 24% of the electricity requirements of the process with a cogeneration (cogen) system (Figure S7). The additional heat is treated as a credit for the LCA. Details of the cogen system can be found in the SI. The combusted methane in the cogeneration system produces extra CO_2_ in a flue gas stream which can either be captured or released to the environment. The post combustion flue gas stream is diluted in N_2_ which makes the CO_2_ difficult to separate. Therefore, this CO_2_ capture is only considered in the Blue PV FGC scenario models. Details are given in supplemental information.

**Table:** Blue process input summary

Section	Technosphere Flow	Quantity (per kg NH_3_)	
		Blue PV	Blue PV FGC
Feeds	Natural Gas	1.2 m3	
	Water	1.76 kg	
	Methanol	0.07 kg	
Utilities	Electricity (including cogen)	0.23 kWh	0.31kWh
	Refrigeration Electricity	1.07 kWh	
	Cooling Water	308.65 kg	368.65 kg
	Heat (including cogen)	−2.2 MJ	0.3 MJ
Infrastructure	CCS Transportation and Storage	1.61 kg stored	2.17 kg stored
	Facility	5.25e-10 units [4]	5.38e-10 units
	Battery (PV scenario)	1.28e-4 kWh capacity	

#### Scenarios and sensitivities

Table 1 summarizes the scenarios included in the LCA. Table 2 summarizes the base scenario parameters and the sensitivity analysis ranges used.

For the blue process, a sensitivity analysis is performed on the natural gas supply chain leakage and on the CCS transportation distance. Emphasis is placed on the natural gas supply chain due to literature indicating large uncertainty and variability in the leakage rates^20^^,^^51^ and a large climate impact coming from methane leakage.^18^^,^^19^

The natural gas supply chain is separated into production and transportation to shed light on different parts of the supply chain and to help make decisions on how far from the natural gas production site a blue ammonia production facility should be placed.

Regarding the sensitivity on the CCS supply chain, distance from CO_2_ storage site is the only parameter varied because compared to methane leakage, literature does not indicate large uncertainty on the CO_2_ leakage rate.^69^ The sensitivity on distance from CO_2_ storage site also helps as a comparison to the natural gas transportation distance.

For the green process, sensitivities are performed on potential technological improvements and on average irradiation to generalize the PV results beyond Saudi Arabia. Bauer et al.^70^ state that out of all renewables, PV is expected to have the largest reduction in GHG emissions due to increased efficiencies in manufacturing and in the PV cells themselves. Frischknecht et al.^71^ carried out a prospective LCA including various environmental impact categories and different scenarios for percent reductions from today’s impacts. These projections are not included in the sensitivity analysis since not all impact categories evaluated in this study were included in the projections. However, it is worth mentioning that improvements in PV supply chains are expected across many impact categories.

To evaluate the energy efficiency of our processes, we carried out a comparison with advanced blue and green ammonia industrial processes^37^ and with other blue ammonia processes from literature^13^^,^^30^^,^^42^ (supporting information Section comparison with advanced blue ammonia processes (LAC/KBR)). While our efficiencies are in line with the process efficiencies from literature, the comparison shows that the green process could improve with higher electrolyzer efficiencies and the blue process could improve with alternative production process concepts.

## Data Availability

All data used in this paper is available in the main text, in the supplementary information, or the sources have been clearly stated. Any additional information required to reanalyze the data reported in this paper are available from the lead contact upon request.

## Footnotes

Supplemental information can be found online at https://doi.org/10.1016/j.isci.2023.107389.
