# Supplementary material for: Blue and green ammonia production: A techno-economic and life cycle assessment perspective
Source: iScience. 2023 Jul 14;26(8):107389. doi: 10.1016/j.isci.2023.107389 (PMC10404734; doi:10.1016/j.isci.2023.107389)
Supplement: Document S1. Figures S1–S7 and Tables S1–S13 [file mmc1.pdf]

**iScience, Volume *26***

## Supplemental information

# Blue and green ammonia production: A techno-economic and life cycle assessment perspective

**Patricia Mayer, Adrian Ramirez, Giuseppe Pezzella, Benedikt Winter, S. Mani Sarathy, Jorge Gascon, and André Bardow**

## BENEFITS OF PROCESS INTEGRATION

The integrated Steam Methane Reforming-Haber Bosch (SMR-HB) blue ammonia production process presents benefits relative to a separated process considering blue hydrogen production from SMR followed by ammonia production from HB. For instance, in the integrated process, air is fed to the SMR part of the process to provide the heat of reaction by partial oxidation of the reagents [1]. The air also provides nitrogen to the HB part of the process in the exact stoichiometric amount needed for ammonia production. As a result, the integrated SMR-HB process does not require a separate nitrogen feed, as would be needed if the hydrogen and ammonia production steps are considered separately [2]. Additionally, the integrated process allows for waste heat from the SMR part of the process to be used in the HB section. The high temperatures needed for SMR lead to substantial amounts of waste heat, which is typically used to generate steam for driving compressors [1].

To demonstrate the benefits of process integration, we compare the Power-to-X efficiency of the integrated blue ammonia process with an equivalent process separating blue hydrogen and ammonia production (**Section** **Power-to-What?**). Blue hydrogen production is modeled using the same process simulation as for blue ammonia without considering the HB portion (**Figure S6**). The subsequent ammonia production is modelled using the same process simulation as for green ammonia (**Figure S5**), with blue hydrogen as a feed. This approach allows us to obtain the most direct comparison between a separated process and our integrated blue ammonia process.

Our blue hydrogen product stream is represented by stream S29 in **Figure S6.** This stream contains 23%(mol) nitrogen and 9%(mol) $CH_4$ and therefore some energy of separation is required to purify the hydrogen. We consider the same amount of hydrogen and $CH_4$ in the PURGE2 stream for the blue hydrogen process as for the blue ammonia process in order to maintain equal contributions from the cogeneration system in the comparison. Therefore, in the separation of stream S29, we do not recover 100% of the hydrogen but rather do a mass balance with stream S29 and stream PURGE2. The nitrogen content in stream S29 is assumed to be mixed with the PURGE2 stream to be sent to the cogeneration system. **Figure S1** shows the composition of the streams resulting from the hypothetical separation.

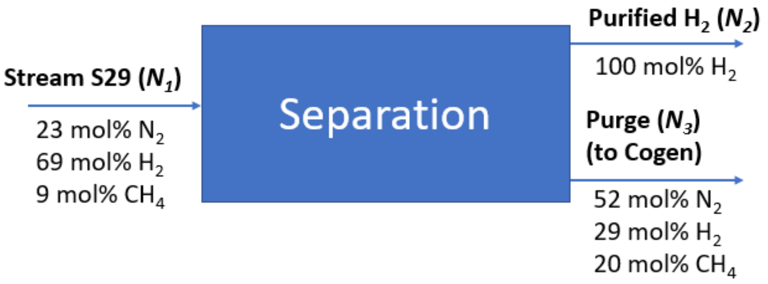


**Figure S1. [Hypothetical separation of hydrogen stream]** Composition of streams resulting from the hypothetical separation of stream S29 to obtain a purified hydrogen stream. $N_i$ refers to the mole flows of each of the streams. Related to **Section** **Power-to-What?**.

To calculate the energy of separation, we first calculate the theoretical minimum work required to separate the hydrogen from the other components in stream S29, following the approach in [3] (***Equation 1***). $W_{min}$ refers to the minimum theoretical work, $N_i$ refers to the molar flow rates of the streams depicted in **Figure S1,** and $X_{j,k}$ refers to the mole fraction of component *k* in stream *j*.

$$W_{min} = -RT(N_1 \sum_{k=1..n} X_{1,k} lnX_{1,k} - N_2 \sum_{k=1..n} X_{2,k} lnX_{2,k} - N_3 \sum_{k=1..n} X_{3,k} lnX_{3,k}) \qquad \text{(Equation 1)}$$

We then calculate the actual work, $W_{actual}$, by considering a second law efficiency of 15% for $H_2$ clean-up from [4]. The calculated separation work amounts to 1.45 kWh/kg $H_2$.

## FIGURES AND TABLES

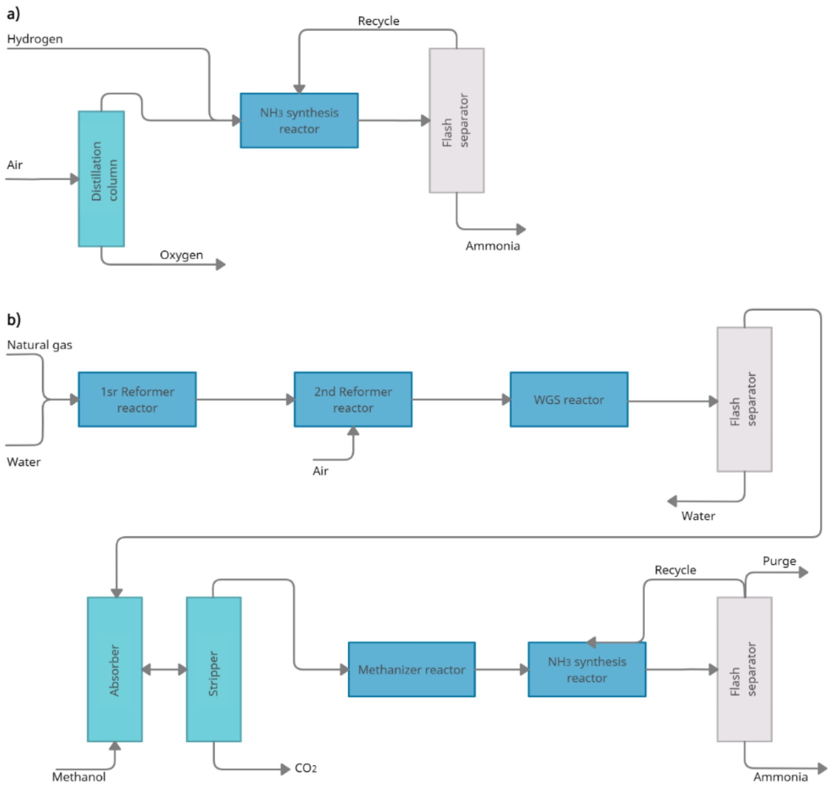


**Figure S2. [Process block flow diagrams]** Block flow diagrams of the two ammonia production processes. a) green, b) blue. Related to STAR Methods.

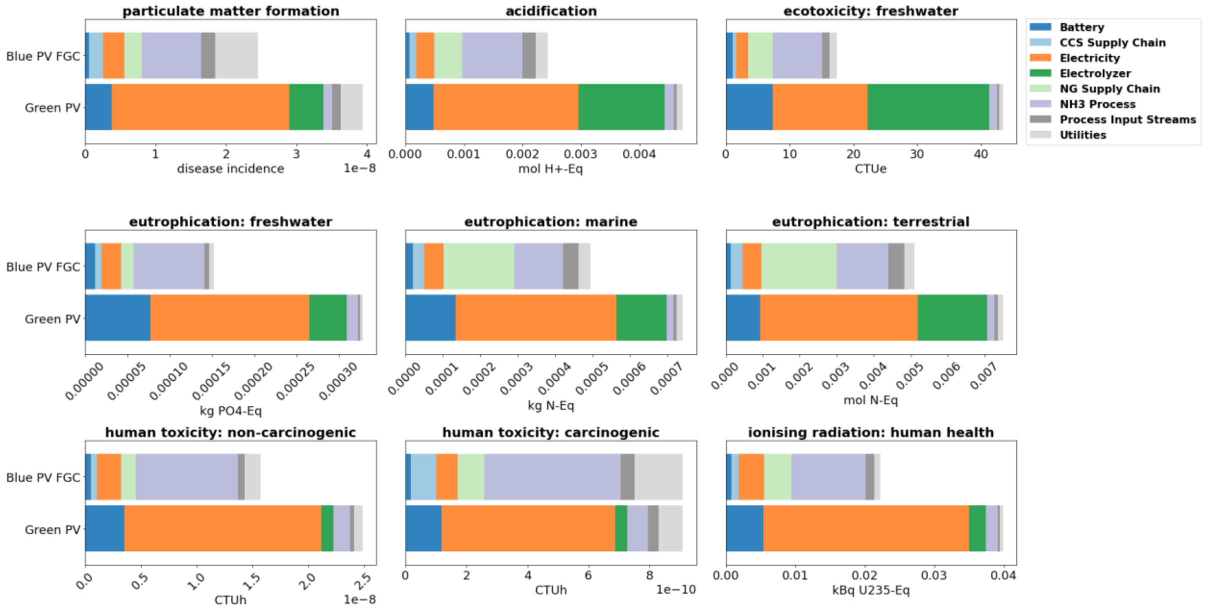


**Figure S3. [Remaining impact categories not discussed in the main text]** Remaining impact categories besides those discussed in **Section** **Environmental Impacts beyond Climate Change.**

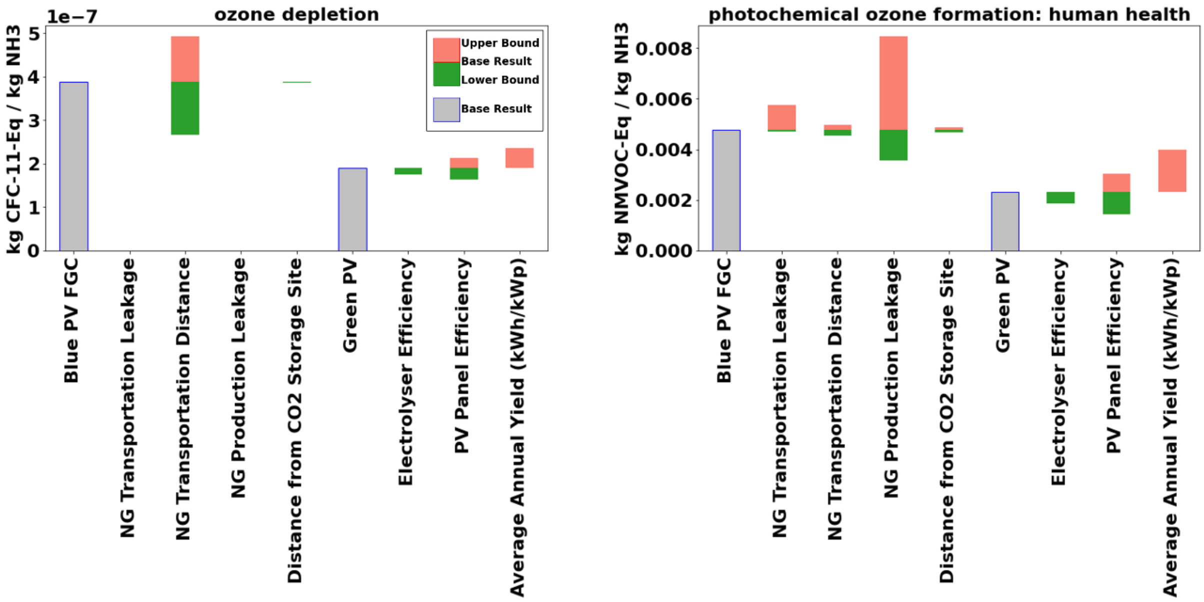


**Figure S4. [Sensitivity analysis results for impact categories sensitive to modelling inputs]** Sensitivity analysis for impact categories sensitive to modelling inputs. Sensitivity ranges are provided in **Section Scenarios and Sensitivities**. Related to **Figure 4**.

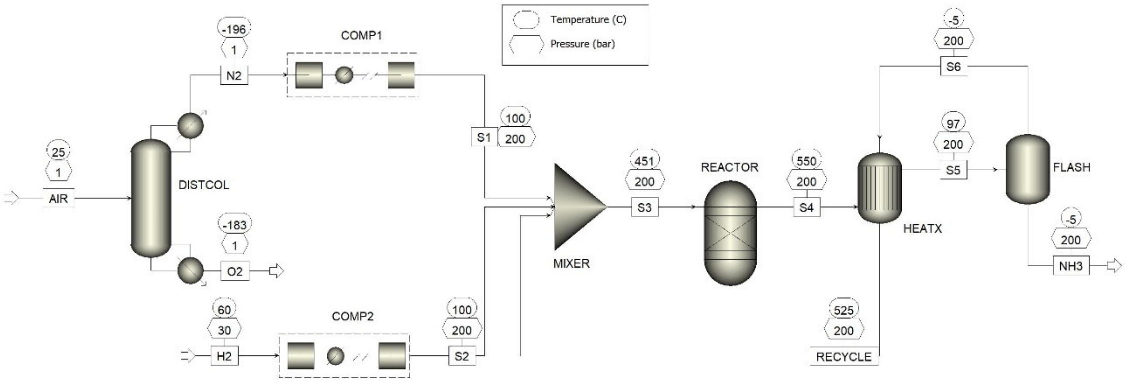


**Figure S5. [Green process Aspen Plus flowsheet]** Aspen Plus flowsheet of the green NH3 process. Related to STAR Methods.

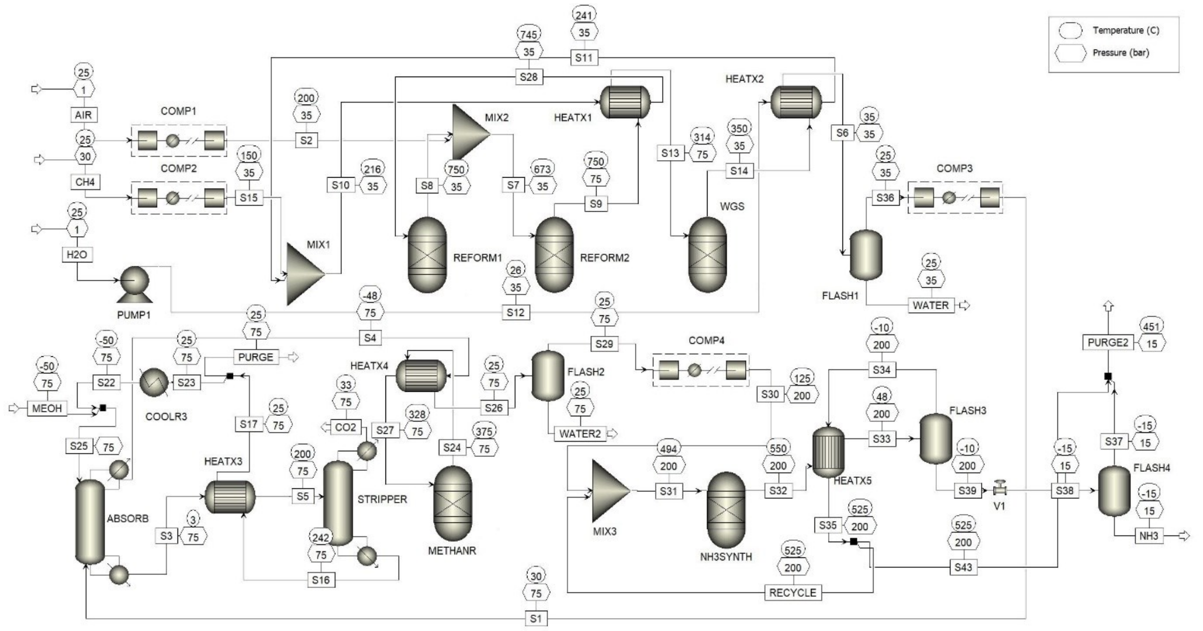


**Figure S6. [Blue process Aspen Plus flowsheet]** Aspen Plus flowsheet of the blue $NH_3$ process. Related to STAR Methods.

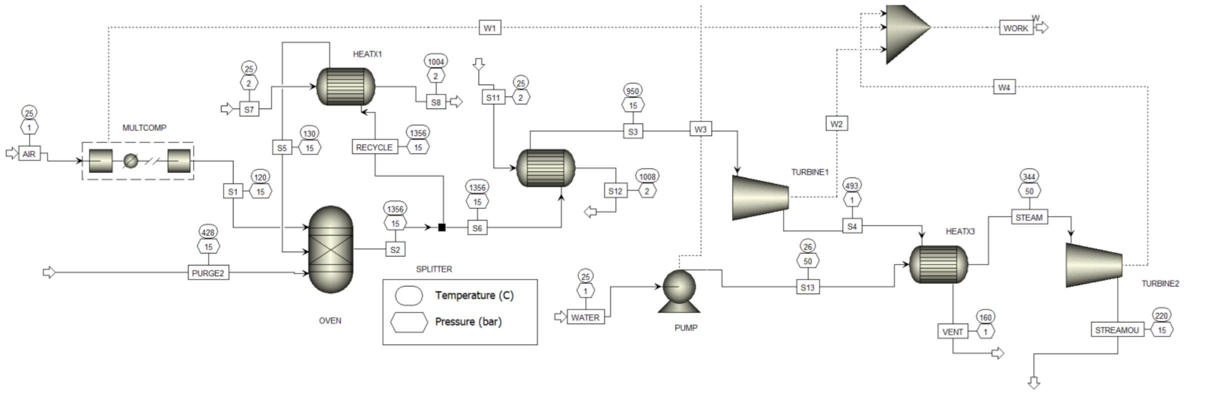


**Figure S7. [Blue process cogeneration system process Aspen Plus flowsheet]** Aspen Plus flowsheet of the cogeneration process. Related to STAR Methods.

**Table S1.** Survey of the state-of-the-art LCA for ammonia synthesis. Related to **Introduction**.

| Location | Method | Case | GWP | Ref |
|---|---|---|---|---|
| Germany | ReCIPe (updated version of CML and Eco-Indicator 99) | SMR + Cryogenic ASU+ HB +electricity grid mix + Chemical Absorption by MDEA+ Electricity grid mix | 2.79 | 2 |
| Germany | ReCIPe (updated version of CML and Eco-Indicator 99) | SMR + Cryogenic ASU+ HB +electricity grid mix + Chemical Absorption by chilled $NH_3$+ Electricity grid mix | 2.80 | 2 |
| Germany | ReCIPe (updated version of CML and Eco-Indicator 99) | Chemical Looping for $N_2$, H2 and AGR + HB + Electricity grid mix | 0.37 | 2 |
| Germany | ReCIPe (updated version of CML and Eco-Indicator 99) | Cryogenic ASU+ Water Electrolysis + HB + Electricity grid mix | 6.72 | 2 |
| Germany | ReCIPe (updated version of CML and Eco-Indicator 99) | Cryogenic ASU+ Water Electrolysis + HB + Renewable electricity grid mix | 0.15 | 2 |
| South Central U.S.A. | GREET 2018 Model (Cradle to Gate) | SMR+ CCS + Cryogenic ASU + HB + Electricity grid mix | 1.00 | 5 |
| South Central U.S.A. | GREET 2018 Model (Cradle to Gate) | SMR+ CCS + Cryogenic ASU + Electrochemical Method + Electricity grid mix | 9.80 | 5 |
| South Central U.S.A. | GREET 2018 Model (Cradle to Gate) | Electrolysis $H_2$ + HB + Cryogenic ASU + Electricity grid mix | 2.75 | 5 |
| South Central U.S.A. | GREET 2018 Model (Cradle to Gate) | Electrolysis $H_2$ + Electrochemical Method + Cryogenic ASU + Electricity grid mix | 3.00 | 5 |
| Not Declared* | CML 2001 (Cradle to Grave) | Nuclear High Temperature electrolysis + Cryogenic ASU+ HB + Electricity grid mix using Nuclear High Temperature Electrolysis | 0.42 | 6 |
| U.S.A. | GREET 2019 Model (Cradle to Gate) | SMR+Cryogenic ASU + HB | 2.55 | 7 |
| North-East U.S.A | GREET 2019 Model (Cradle to Gate) | Low-Temperature Electrolysis + Cryogenic ASU + HB + Northeast Power Coordinating Council grid mix | 2.50 | 7 |
| U.S.A. | GREET 2019 Model (Cradle to Gate) | Low-Temperature Electrolysis + Cryogenic ASU + HB + Average U.S.A. national grid | 4.50 | 7 |
| Midwest U.S.A. | GREET 2019 Model (Cradle to Gate) | Low-Temperature Electrolysis + Cryogenic ASU + HB + Midwest Reliability Organization grid | 6.00 | 7 |
| U.S.A. | GREET 2019 Model (Cradle to Gate) | Low-Temperature Electrolysis + Cryogenic ASU + HB + Wind or Solar Electricity | 0.22 | 7 |
| U.S.A. | GREET 2019 Model (Cradle to Gate) | High-Temperature Electrolysis + Cryogenic ASU + HB + Wind or Solar Electricity | 0.25 | 7 |

| | | | | |
|---|---|---|---|---|
| Country mix | ReCIPe (Cradle to Gate) | Thermal catalytic cracking of LNG with solar tower | 0.62 | 8 |
| Algeria (Annaba) | Global Emission Model for Integrated Systems software (Cradle to Gate) | SMR+HB+Algerian Electricity national grid | 1.44 | 9 |

**Table S2.** Description of the different utilities used in the process modeling. Related to STAR Methods.

| **Utility ID** | | CRIO | ELECTRIC | REFRIG | STEAM | WATER | GASHEAT |
|---|---|---|---|---|---|---|---|
| **Heating/Cooling value** | cal/gm | -0.32 | | -0.96 | 523.52 | -4.99 | 143.31 |
| **Electricity price** | $/kWhr | | 0.08 | | | | |
| **Energy price** | $/cal | 3.73E-08 | | 1.15E-08 | 7.95E-09 | 8.88E-10 | 0.00 |
| **Inlet temperature** | C | -270.00 | | -25.00 | 125.00 | 20.00 | 1000.00 |
| **Outlet temperature** | C | -269.00 | | -24.00 | 124.00 | 25.00 | 800.00 |

**Table S3.** Stream composition of the green process. Related to STAR Methods.

| **Stream Name** | AIR | H2 | N2 | NH3 | O2 | RECYCLE | S1 | S2 | S3 | S4 | S5 | S6 |
|---|---|---|---|---|---|---|---|---|---|---|---|---|
| **From** | | | DISTCOL | FLASH | DISTCOL | HEATX | COMP1 | COMP2 | MIXER | REACTOR | HEATX | FLASH |
| **To** | DISTCOL | COMP2 | COMP1 | | | MIXER | MIXER | MIXER | REACTOR | HEATX | FLASH | HEATX |
| **Temperature (C)** | 25.00 | 60.00 | -195.71 | -5.00 | -182.76 | 525.00 | 100.00 | 100.00 | 451.22 | 550.00 | 97.19 | -5.00 |
| **Pressure (bar)** | 1.00 | 30.00 | 1.00 | 200.00 | 1.00 | 200.00 | 200.00 | 200.00 | 200.00 | 200.00 | 200.00 | 200.00 |
| **Mole Flows (kmol/h)** | 5000.00 | 11850.00 | 3950.00 | 7930.73 | 1050.00 | 73935.00 | 3950.00 | 11850.00 | 89734.96 | 81866.39 | 81866.39 | 73935.00 |
| **O2** | 1050.00 | 0.00 | 0.00 | 0.00 | 1050.00 | 0.00 | 0.00 | 0.00 | 0.00 | 0.00 | 0.00 | 0.00 |
| **N2** | 3950.00 | 0.00 | 3950.00 | 14.50 | 0.00 | 16515.79 | 3950.00 | 0.00 | 20465.79 | 16531.50 | 16531.50 | 16515.79 |
| **H2** | 0.00 | 11850.00 | 0.00 | 47.78 | 0.00 | 54976.80 | 0.00 | 11850.00 | 66826.78 | 55023.92 | 55023.92 | 54976.80 |
| **NH3** | 0.00 | 0.00 | 0.00 | 7868.45 | 0.00 | 2442.40 | 0.00 | 0.00 | 2442.39 | 10310.97 | 10310.97 | 2442.40 |
| **CO** | 0.00 | 0.00 | 0.00 | 0.00 | 0.00 | 0.00 | 0.00 | 0.00 | 0.00 | 0.00 | 0.00 | 0.00 |
| **CO2** | 0.00 | 0.00 | 0.00 | 0.00 | 0.00 | 0.00 | 0.00 | 0.00 | 0.00 | 0.00 | 0.00 | 0.00 |
| **CH4** | 0.00 | 0.00 | 0.00 | 0.00 | 0.00 | 0.00 | 0.00 | 0.00 | 0.00 | 0.00 | 0.00 | 0.00 |
| **H2O** | 0.00 | 0.00 | 0.00 | 0.00 | 0.00 | 0.00 | 0.00 | 0.00 | 0.00 | 0.00 | 0.00 | 0.00 |
| **Mole Fractions** | | | | | | | | | | | | |
| **O2** | 0.21 | 0.00 | 0.00 | 0.00 | 1.00 | 0.00 | 0.00 | 0.00 | 0.00 | 0.00 | 0.00 | 0.00 |
| **N2** | 0.79 | 0.00 | 1.00 | 0.00 | 0.00 | 0.22 | 1.00 | 0.00 | 0.23 | 0.20 | 0.20 | 0.22 |
| **H2** | 0.00 | 1.00 | 0.00 | 0.01 | 0.00 | 0.74 | 0.00 | 1.00 | 0.74 | 0.67 | 0.67 | 0.74 |
| **NH3** | 0.00 | 0.00 | 0.00 | 0.99 | 0.00 | 0.03 | 0.00 | 0.00 | 0.03 | 0.13 | 0.13 | 0.03 |
| **CO** | 0.00 | 0.00 | 0.00 | 0.00 | 0.00 | 0.00 | 0.00 | 0.00 | 0.00 | 0.00 | 0.00 | 0.00 |
| **CO2** | 0.00 | 0.00 | 0.00 | 0.00 | 0.00 | 0.00 | 0.00 | 0.00 | 0.00 | 0.00 | 0.00 | 0.00 |
| **CH4** | 0.00 | 0.00 | 0.00 | 0.00 | 0.00 | 0.00 | 0.00 | 0.00 | 0.00 | 0.00 | 0.00 | 0.00 |
| **H2O** | 0.00 | 0.00 | 0.00 | 0.00 | 0.00 | 0.00 | 0.00 | 0.00 | 0.00 | 0.00 | 0.00 | 0.00 |

**Table S4.1.** Stream composition of the blue process. Related to STAR Methods.

| Stream Name | AIR | CH4 | CO2 | H2O | MEOH | NH3 | PURGE | PURGE2 | RECYCLE | S1 | S2 |
|---|---|---|---|---|---|---|---|---|---|---|---|
| From | | | STRIPPER | | | FLASH4 | B2 | B6 | B1 | COMP3 | COMP1 |
| To | COMP1 | COMP2 | | PUMP1 | B3 | | | | MIX3 | ABSORB | MIX2 |
| Temperature (C) | 25,00 | 25,00 | 92,52 | 25,00 | -50,00 | -16,28 | 25,00 | 428,23 | 525,03 | 30,00 | 200,00 |
| Pressure (bar) | 1,00 | 30,00 | 75,00 | 1,00 | 75,00 | 15,00 | 75,00 | 15,00 | 200,00 | 75,00 | 35,00 |
| Mole Flows (kmol/h) | 6000,00 | 6000,00 | 5000,00 | 12000,00 | 275,74 | 7224,37 | 43,25 | 5315,83 | 170287,12 | 26079,11 | 6000,00 |
| O2 | 1260,00 | 0,00 | 0,00 | 0,00 | 0,00 | 0,00 | 0,00 | 0,00 | 0,00 | 0,00 | 1260,00 |
| N2 | 4740,00 | 0,00 | 242,75 | 0,00 | 0,00 | 7,81 | 0,00 | 725,50 | 23893,32 | 4739,81 | 4740,00 |
| H2 | 0,00 | 0,00 | 210,89 | 0,00 | 0,00 | 2,32 | 0,00 | 2519,86 | 92363,64 | 15321,26 | 0,00 |
| NH3 | 0,00 | 0,00 | 0,00 | 0,00 | 0,00 | 7085,98 | 0,00 | 321,99 | 5694,41 | 0,00 | 0,00 |
| CO | 0,00 | 0,00 | 27,91 | 0,00 | 0,00 | 0,00 | 0,00 | 0,00 | 0,00 | 510,80 | 0,00 |
| CO2 | 0,00 | 0,00 | 4051,35 | 0,00 | 0,00 | 0,00 | 0,00 | 0,00 | 0,00 | 4067,25 | 0,00 |
| CH4 | 0,00 | 6000,00 | 195,98 | 0,00 | 0,00 | 117,34 | 0,00 | 1748,46 | 48335,46 | 1411,66 | 0,00 |
| H2O | 0,00 | 0,00 | 25,62 | 12000,00 | 0,00 | 10,92 | 13,43 | 0,01 | 0,29 | 28,33 | 0,00 |
| MEOH | 0,00 | 0,00 | 245,50 | 0,00 | 275,74 | 0,00 | 29,81 | 0,00 | 0,00 | 0,00 | 0,00 |
| Mole Fractions | | | | | | | | | | | |
| O2 | 0,21 | 0,00 | 0,00 | 0,00 | 0,00 | 0,00 | 0,00 | 0,00 | 0,00 | 0,00 | 0,21 |
| N2 | 0,79 | 0,00 | 0,05 | 0,00 | 0,00 | 0,00 | 0,00 | 0,14 | 0,14 | 0,18 | 0,79 |
| H2 | 0,00 | 0,00 | 0,04 | 0,00 | 0,00 | 0,00 | 0,00 | 0,47 | 0,54 | 0,59 | 0,00 |
| NH3 | 0,00 | 0,00 | 0,00 | 0,00 | 0,00 | 0,99 | 0,00 | 0,06 | 0,03 | 0,00 | 0,00 |
| CO | 0,00 | 0,00 | 0,01 | 0,00 | 0,00 | 0,00 | 0,00 | 0,00 | 0,00 | 0,02 | 0,00 |
| CO2 | 0,00 | 0,00 | 0,81 | 0,00 | 0,00 | 0,00 | 0,00 | 0,00 | 0,00 | 0,16 | 0,00 |
| CH4 | 0,00 | 1,00 | 0,04 | 0,00 | 0,00 | 0,01 | 0,00 | 0,33 | 0,28 | 0,05 | 0,00 |
| H2O | 0,00 | 0,00 | 0,01 | 1,00 | 0,00 | 0,00 | 0,31 | 0,00 | 0,00 | 0,00 | 0,00 |
| MEOH | 0,00 | 0,00 | 0,05 | 0,00 | 1,00 | 0,00 | 0,69 | 0,00 | 0,00 | 0,00 | 0,00 |

**Table S4.2.** Stream composition of the blue process. Related to STAR Methods.

| Stream Name | S3 | S4 | S5 | S6 | S7 | S8 | S9 | S10 | S11 | S12 | S13 | S14 |
|---|---|---|---|---|---|---|---|---|---|---|---|---|
| **From** | ABSORB | ABSORB | HEATX3 | HEATX2 | MIX2 | REFORM1 | REFORM2 | MIX1 | HEATX2 | PUMP1 | HEATX1 | WGS |
| **To** | HEATX3 | HEATX4 | STRIPPER | FLASH1 | REFORM2 | MIX2 | HEATX1 | HEATX1 | MIX1 | HEATX2 | WGS | HEATX2 |
| **Temperature (C)** | 3,00 | -48,00 | 198,51 | 35,00 | 659,86 | 750,00 | 865,00 | 216,69 | 244,83 | 25,58 | 464,61 | 350,00 |
| **Pressure (bar)** | 75,00 | 75,00 | 75,00 | 35,00 | 35,00 | 35,00 | 35,00 | 35,00 | 35,00 | 35,00 | 35,00 | 35,00 |
| **Mole Flows (kmol/h)** | 48294,85 | 21322,33 | 48294,85 | 31916,26 | 28712,40 | 22712,40 | 31916,26 | 18000,00 | 12000,00 | 12000,00 | 31916,26 | 31916,26 |
| **O2** | 0,00 | 0,00 | 0,00 | 0,00 | 1260,00 | 0,00 | 0,00 | 0,00 | 0,00 | 0,00 | 0,00 | 0,00 |
| **N2** | 242,75 | 4497,07 | 242,75 | 4740,00 | 4740,00 | 0,00 | 4740,00 | 0,00 | 0,00 | 0,00 | 4740,00 | 4740,00 |
| **H2** | 210,89 | 15110,37 | 210,89 | 15321,69 | 8398,36 | 8398,36 | 12670,17 | 0,00 | 0,00 | 0,00 | 12670,17 | 15321,69 |
| **NH3** | 0,00 | 0,00 | 0,00 | 0,00 | 0,00 | 0,00 | 0,00 | 0,00 | 0,00 | 0,00 | 0,00 | 0,00 |
| **CO** | 27,91 | 482,89 | 27,91 | 510,82 | 1026,44 | 1026,44 | 3162,34 | 0,00 | 0,00 | 0,00 | 3162,34 | 510,82 |
| **CO2** | 4051,82 | 15,90 | 4051,82 | 4077,30 | 1329,76 | 1329,76 | 1425,79 | 0,00 | 0,00 | 0,00 | 1425,79 | 4077,30 |
| **CH4** | 195,98 | 1215,68 | 195,98 | 1411,87 | 3643,80 | 3643,80 | 1411,87 | 6000,00 | 0,00 | 0,00 | 1411,87 | 1411,87 |
| **H2O** | 13462,16 | 0,00 | 13462,16 | 5854,57 | 8314,04 | 8314,04 | 8506,09 | 12000,00 | 12000,00 | 12000,00 | 8506,09 | 5854,57 |
| **MEOH** | 30103,33 | 0,42 | 30103,33 | 0,00 | 0,00 | 0,00 | 0,00 | 0,00 | 0,00 | 0,00 | 0,00 | 0,00 |
| **Mole Fractions** | | | | | | | | | | | | |
| **O2** | 0,00 | 0,00 | 0,00 | 0,00 | 0,04 | 0,00 | 0,00 | 0,00 | 0,00 | 0,00 | 0,00 | 0,00 |
| **N2** | 0,01 | 0,21 | 0,01 | 0,15 | 0,17 | 0,00 | 0,15 | 0,00 | 0,00 | 0,00 | 0,15 | 0,15 |
| **H2** | 0,00 | 0,71 | 0,00 | 0,48 | 0,29 | 0,37 | 0,40 | 0,00 | 0,00 | 0,00 | 0,40 | 0,48 |
| **NH3** | 0,00 | 0,00 | 0,00 | 0,00 | 0,00 | 0,00 | 0,00 | 0,00 | 0,00 | 0,00 | 0,00 | 0,00 |
| **CO** | 0,00 | 0,02 | 0,00 | 0,02 | 0,04 | 0,05 | 0,10 | 0,00 | 0,00 | 0,00 | 0,10 | 0,02 |
| **CO2** | 0,08 | 0,00 | 0,08 | 0,13 | 0,05 | 0,06 | 0,04 | 0,00 | 0,00 | 0,00 | 0,04 | 0,13 |
| **CH4** | 0,00 | 0,06 | 0,00 | 0,04 | 0,13 | 0,16 | 0,04 | 0,33 | 0,00 | 0,00 | 0,04 | 0,04 |
| **H2O** | 0,28 | 0,00 | 0,28 | 0,18 | 0,29 | 0,37 | 0,27 | 0,67 | 1,00 | 1,00 | 0,27 | 0,18 |
| **MEOH** | 0,62 | 0,00 | 0,62 | 0,00 | 0,00 | 0,00 | 0,00 | 0,00 | 0,00 | 0,00 | 0,00 | 0,00 |

**Table S4.3.** Stream composition of the blue process.

| Stream Name | S15 | S16 | S17 | S22 | S23 | S24 | S25 | S26 | S27 | S28 | S29 | S30 |
|---|---|---|---|---|---|---|---|---|---|---|---|---|
| From | COMP2 | STRIPPER | HEATX3 | COOLR3 | B2 | METHANR | B3 | HEATX4 | HEATX4 | HEATX1 | FLASH2 | COMP4 |
| To | MIX1 | HEATX3 | B2 | B3 | COOLR3 | HEATX4 | ABSORB | FLASH2 | METHANR | REFORM1 | COMP4 | MIX3 |
| Temperature (C) | 150,00 | 241,83 | 25,00 | -50,00 | 25,00 | 375,00 | | 25,00 | 327,39 | 745,00 | 25,00 | 125,00 |
| Pressure (bar) | 35,00 | 75,00 | 75,00 | 75,00 | 75,00 | 75,00 | 75,00 | 75,00 | 75,00 | 35,00 | 75,00 | 200,00 |
| Mole Flows (kmol/h) | 6000,00 | 43246,91 | 43246,91 | 43203,66 | 43203,66 | 20324,74 | 43538,07 | 20324,74 | 21322,33 | 18000,00 | 19820,35 | 19820,35 |
| O2 | 0,00 | 0,00 | 0,00 | 0,00 | 0,00 | 0,00 | 0,00 | 0,00 | 0,00 | 0,00 | 0,00 | 0,00 |
| N2 | 0,00 | 0,00 | 0,00 | 0,00 | 0,00 | 4497,07 | 0,00 | 4497,07 | 4497,07 | 0,00 | 4497,03 | 4497,03 |
| H2 | 0,00 | 0,00 | 0,00 | 0,00 | 0,00 | 13598,07 | 0,00 | 13598,07 | 15110,37 | 0,00 | 13597,98 | 13597,98 |
| NH3 | 0,00 | 0,00 | 0,00 | 0,00 | 0,00 | 0,00 | 0,00 | 0,00 | 0,00 | 0,00 | 0,00 | 0,00 |
| CO | 0,00 | 0,00 | 0,00 | 0,00 | 0,00 | 0,00 | 0,00 | 0,00 | 482,89 | 0,00 | 0,00 | 0,00 |
| CO2 | 0,00 | 0,48 | 0,48 | 0,48 | 0,48 | 0,00 | 0,48 | 0,00 | 15,90 | 0,00 | 0,00 | 0,00 |
| CH4 | 6000,00 | 0,00 | 0,00 | 0,00 | 0,00 | 1714,48 | 0,00 | 1714,48 | 1215,68 | 6000,00 | 1714,42 | 1714,42 |
| H2O | 0,00 | 13434,30 | 13434,30 | 13420,86 | 13420,86 | 514,70 | 13433,84 | 514,70 | 0,00 | 12000,00 | 10,73 | 10,73 |
| MEOH | 0,00 | 29812,13 | 29812,13 | 29782,32 | 29782,32 | 0,42 | 30103,76 | 0,42 | 0,42 | 0,00 | 0,19 | 0,19 |
| Mole Fractions | | | | | | | | | | | | |
| O2 | 0,00 | 0,00 | 0,00 | 0,00 | 0,00 | 0,00 | 0,00 | 0,00 | 0,00 | 0,00 | 0,00 | 0,00 |
| N2 | 0,00 | 0,00 | 0,00 | 0,00 | 0,00 | 0,22 | 0,00 | 0,22 | 0,21 | 0,00 | 0,23 | 0,23 |
| H2 | 0,00 | 0,00 | 0,00 | 0,00 | 0,00 | 0,67 | 0,00 | 0,67 | 0,71 | 0,00 | 0,69 | 0,69 |
| NH3 | 0,00 | 0,00 | 0,00 | 0,00 | 0,00 | 0,00 | 0,00 | 0,00 | 0,00 | 0,00 | 0,00 | 0,00 |
| CO | 0,00 | 0,00 | 0,00 | 0,00 | 0,00 | 0,00 | 0,00 | 0,00 | 0,02 | 0,00 | 0,00 | 0,00 |
| CO2 | 0,00 | 0,00 | 0,00 | 0,00 | 0,00 | 0,00 | 0,00 | 0,00 | 0,00 | 0,00 | 0,00 | 0,00 |
| CH4 | 1,00 | 0,00 | 0,00 | 0,00 | 0,00 | 0,08 | 0,00 | 0,08 | 0,06 | 0,33 | 0,09 | 0,09 |
| H2O | 0,00 | 0,31 | 0,31 | 0,31 | 0,31 | 0,03 | 0,31 | 0,03 | 0,00 | 0,67 | 0,00 | 0,00 |
| MEOH | 0,00 | 0,69 | 0,69 | 0,69 | 0,69 | 0,00 | 0,69 | 0,00 | 0,00 | 0,00 | 0,00 | 0,00 |

**Table S4.4.** Stream composition of the blue process. Related to STAR Methods.

| **Stream Name** | S31 | S32 | S33 | S34 | S35 | S36 | S37 | S38 | S39 | S43 | WATER | WATER2 |
|---|---|---|---|---|---|---|---|---|---|---|---|---|
| **From** | MIX3 | NH3SYNTH | HEATX5 | FLASH3 | HEATX5 | FLASH1 | FLASH4 | V1 | FLASH3 | B1 | FLASH1 | FLASH2 |
| **To** | NH3SYNTH | HEATX5 | FLASH3 | HEATX5 | B1 | COMP3 | B6 | FLASH4 | V1 | B6 | | |
| **Temperature (C)** | 489,47 | 550,00 | 52,45 | -10,00 | 525,00 | 25,00 | -16,28 | -16,28 | -10,00 | 525,03 | 25,00 | 25,00 |
| **Pressure (bar)** | 200,00 | 200,00 | 200,00 | 200,00 | 200,00 | 35,00 | 15,00 | 15,00 | 200,00 | 200,00 | 35,00 | 75,00 |
| **Mole Flows (kmol/h)** | 190107,47 | 182685,78 | 182685,78 | 174779,26 | 174779,26 | 26079,11 | 949,49 | 8173,86 | 8173,86 | 4366,34 | 5837,15 | 504,39 |
| **O2** | 0,00 | 0,00 | 0,00 | 0,00 | 0,00 | 0,00 | 0,00 | 0,00 | 0,00 | 0,00 | 0,00 | 0,00 |
| **N2** | 28390,34 | 24679,50 | 24679,50 | 24544,19 | 24544,19 | 4739,81 | 112,85 | 120,67 | 120,67 | 612,65 | 0,19 | 0,04 |
| **H2** | 105961,62 | 94828,90 | 94828,90 | 94752,31 | 94752,31 | 15321,26 | 151,56 | 153,88 | 153,88 | 2368,30 | 0,43 | 0,09 |
| **NH3** | 5694,41 | 13116,10 | 13116,10 | 5846,21 | 5846,21 | 0,00 | 175,98 | 7261,96 | 7261,96 | 146,01 | 0,00 | 0,00 |
| **CO** | 0,00 | 0,00 | 0,00 | 0,00 | 0,00 | 510,80 | 0,00 | 0,00 | 0,00 | 0,00 | 0,02 | 0,00 |
| **CO2** | 0,00 | 0,00 | 0,00 | 0,00 | 0,00 | 4067,25 | 0,00 | 0,00 | 0,00 | 0,00 | 10,06 | 0,00 |
| **CH4** | 50049,87 | 50050,07 | 50050,07 | 49636,25 | 49636,25 | 1411,66 | 509,09 | 626,43 | 626,43 | 1239,37 | 0,21 | 0,06 |
| **H2O** | 11,03 | 11,22 | 11,22 | 0,30 | 0,30 | 28,33 | 0,00 | 10,92 | 10,92 | 0,01 | 5826,24 | 503,97 |
| **MEOH** | 0,19 | 0,00 | 0,00 | 0,00 | 0,00 | 0,00 | 0,00 | 0,00 | 0,00 | 0,00 | 0,00 | 0,23 |
| **Mole Fractions** | | | | | | | | | | | | |
| **O2** | 0,00 | 0,00 | 0,00 | 0,00 | 0,00 | 0,00 | 0,00 | 0,00 | 0,00 | 0,00 | 0,00 | 0,00 |
| **N2** | 0,15 | 0,14 | 0,14 | 0,14 | 0,14 | 0,18 | 0,12 | 0,01 | 0,01 | 0,14 | 0,00 | 0,00 |
| **H2** | 0,56 | 0,52 | 0,52 | 0,54 | 0,54 | 0,59 | 0,16 | 0,02 | 0,02 | 0,54 | 0,00 | 0,00 |
| **NH3** | 0,03 | 0,07 | 0,07 | 0,03 | 0,03 | 0,00 | 0,19 | 0,89 | 0,89 | 0,03 | 0,00 | 0,00 |
| **CO** | 0,00 | 0,00 | 0,00 | 0,00 | 0,00 | 0,02 | 0,00 | 0,00 | 0,00 | 0,00 | 0,00 | 0,00 |
| **CO2** | 0,00 | 0,00 | 0,00 | 0,00 | 0,00 | 0,16 | 0,00 | 0,00 | 0,00 | 0,00 | 0,00 | 0,00 |
| **CH4** | 0,26 | 0,27 | 0,27 | 0,28 | 0,28 | 0,05 | 0,54 | 0,08 | 0,08 | 0,28 | 0,00 | 0,00 |
| **H2O** | 0,00 | 0,00 | 0,00 | 0,00 | 0,00 | 0,00 | 0,00 | 0,00 | 0,00 | 0,00 | 1,00 | 1,00 |
| **MEOH** | 0,00 | 0,00 | 0,00 | 0,00 | 0,00 | 0,00 | 0,00 | 0,00 | 0,00 | 0,00 | 0,00 | 0,00 |

**Table S5.** Survey of the state-of-the-art process simulations for ammonia synthesis. Related to **Section** **Techno-Economic Analysis**.

| **Location** | **Case** | **Heat [$kWh_{th}$/kg]** | **Power [kWh/kg]** | **$NH_3$ production [ton/year]** | **Ref.** |
|---|---|---|---|---|---|
| Taltal, Chile | Cryogenic ASU, Electrolyzer for H2 production, HB | NA | 22 | 35000 | 10 |
| South Central U.S.A. | ASU, polymer electrolyte membrane, HB | NA | 29.7 | 51100 | 5 |
| South Central U.S.A. | ASU, Steam Methane Reforming, electrochemical reactor for NH3 production | NA | 25.2 | 51100 | 5 |
| South Central U.S.A. | ASU, Steam Methane Reforming with CO2 Capture, electrochemical reactor for NH3 production | NA | 26.3 | 51100 | 5 |
| South Central U.S.A | ASU, polymer electrolyte membrane, electrochemical reactor | NA | 32.4 | 51100 | 5 |
| Not Specified | PSA+H2 and O2 combustion, electrolysis with wind power, HB | 1.98 | 11.95 | 2030 | 11 |
| Not Specified | Solid Oxide electrolysis, ASU, HB | NA | 8.46 | 50000 | 12 |
| German Coastal Area | Alkaline Electrolyzer, Mechanical Vapor Compressor, ASU, EHBP, Ammonia Storage System | NA | 9.89 | 109500 | 13 |
| German Coastal Area | PEM Electrolyzer, MVC, ASU, EHBP, Ammonia Storage System | NA | 12.60 | 109500 | 13 |

## METHODS ADDITIONAL INFORMATION

### Process Simulations

Simulations of the green and blue $NH_3$ processes were carried out with steady-state simulation models developed in Aspen Plus® V11 software. The selected property method was Redlich-Kwong-Soave with the Bostton-Mathias modifications. The plant capacity for both processes was set to 1.2 million ton per year with above 99% purity.

The green process was modeled according to the flowsheet in **Figure S5**. The cryogenic air distillation column was simulated using the RadFrac model. The number of trays in the column was fixed to 40 with constant pressure. The feed was introduced to the column above stage 20. Feed pressure was set to 1 bar. The reflux ratio was ser to 2.5. Condenser and reboiler temperatures were set at -195 ºC and -182 ºC respectively. The multistage $N_2$ compressor consist of 3 isentropic compressors with intermediate cooling to 50ºC. The $H_2$ production via electrolyzer was not included in the Aspen simulation boundaries and the $H_2$ was directly fed into the system at 30 bar and 60ºC. The multistage $H_2$ compressor consist of 4 isentropic compressors with intermediate cooling to 50ºC. The efficiency was considered as 0.72. The $NH_3$ synthesis reactor was modeled as a Gibbs equilibrium reactor and operates at 550 ºC and 200 bar. The heat exchanger follows the shortcut model in countercurrent flow direction. The flash separator was based on L-V equilibrium and operates at -5 ºC and 200 bar.

The blue process was modeled according to the flowsheet in **Figure S6**. The primary and secondary reformer reactors operate at 750ºC and 35 bar and 865ºC and 35 bar respectively, and were modeled as a rigorous equilibrium reactor based on stochiometric temperature approach (i.e. Aspen scenario where the temperature & the reaction stoichiometry are known but information on kinetics is not available). The water gas shift reactor operates at 350ºC and 35 bar and was modeled in a similar manner. The methanizer reactor operates at 375ºC and 75 bar and was also modeled as a rigorous equilibrium reactor based on stochiometric temperature approach. The $NH_3$ synthesis reactor was modeled as a Gibbs equilibrium reactor and operates at 550ºC and 200 bar. The below equations were considered for the stochiometric equilibrium reactors:

*Equation 2:* $CH_4 + H_2O \leftrightarrow 3H_2 + CO$

*Equation 3:* $CO + H_2O \leftrightarrow CO_2 + H_2$

*Equation 4:* $2CH_4 + O_2 \leftrightarrow 2CO + 4H_2$

*Equation 5:* $CH_4 + 2O_2 \leftrightarrow CO_2 + 2H_2O$

*Equation 6:* $CO + 3H_2 \leftrightarrow CH_4 + H_2O$

*Equation 7:* $CO_2 + 4H_2 \leftrightarrow CH_4 + 2H_2O$

The $CO_2$ separation was carried out with methanol absorption. The selected properties method was PC-SAFT. The absorption was modeled based on the chemical equilibrium between the absorber and regeneration stripper in a closed cycle. The absorber exhibited 20 stages and operated at 3 °C, while the striper displayed 15 stages with a reflux ratio of 2. The flow of methanol in the absorber was optimized to achieve a methanol/$CO_2$ molar ratio of 7.6. The multistage compressors consist of 2 isentropic compression steps with intermediate cooling to 50ºC. An efficiency of 0.72 was used in the simulations. The flash separators and heat exchangers were modeled in a similar way as in the green process.

The cogeneration process was modeled according to the flowsheet of **Figure S7**. The mixture of the purge of the blue process is compressed up to 15 bar through 3 isentropic compression stages and intermediate cooled to 120 ˚C. The compressed gas is then burned with stoichiometric air in an oven modeled as an adiabatic Gibbs equilibrium reactor, working at 15 bar. A fraction of the exhausted gas (40%) recirculates back to the oven to better control combustion temperature and emissions. Three heat exchangers were modeled in a similar way

to the green process and the hot stream outlet temperature was set to 130 ˚C, 950˚C, and 160˚C. The two turbines' isentropic efficiency is 0.80 while their outlet pressure is 1 bar for the gas turbine and 15 bar for the vapor turbine. The pump has 0.80 isentropic efficiency and brings water pressure up to 50 bar prior to being superheated to 345˚C using the exhausts from the gas turbine.

### Comparison with Advanced Blue Ammonia Processes (LAC/KBR)

We benchmarked our blue process model against the advanced industrial processes Kellogg, Braun & Root (KBR) and the Linde ammonia concept (LAC). We evaluated two energy performance indicators, equivalent thermal efficiency ($\eta_{NH_3eq}$) and equivalent specific consumption ($SC_{eq}$) , as presented by del Pozo and Cloete [14]. Both indicators consider the methane feedstock as lower heating value ($LHV_{NG}$) and the electricity requirements, $\dot{W}_{el}$, by introducing a heat-to-electricity equivalent factor $\eta_{el}$ of 62% representative of a combined cycle power plant. See *Equations 8-10*.

$$\dot{m}_{NG,eq}LHV_{NG} = \dot{m}_{NG}LHV_{NG} + \frac{\dot{W}_{el}}{\eta_{el}} \qquad \text{(Equation 8)}$$

$$\eta_{NH_3eq} = \frac{\dot{m}_{NH_3}LHV_{NH_3}}{\dot{m}_{NG,eq}LHV_{NG}} \qquad \text{(Equation 9)}$$

$$SC_{eq} = \frac{\dot{m}_{NG,eq}LHV_{NG}}{\dot{m}_{NH_3}} \qquad \text{(Equation 10)}$$

**Table S6** shows the performance indicator results for our blue processes in comparison to KBR, LAC, and various processes reported in literature. For completion, we also compare our green process, which uses a 60% electrolyzer efficiency, to the one reported by del Pozo and Cloete [14], which uses a 70% electrolyzer efficiency. We recalculate our performance with a 70% efficiency to align the assumptions.

Regarding our blue process, KBR and LAC processes exhibit a better performance, indicating that our process could be further optimized. However, our blue process is in line with the other literature studies. Regarding our green process, the performance is similar to that of del Pozo and Cloete [14] when considering an efficiency of 70%.

**Table S6:** Energy performance indicator comparison for blue and green ammonia [14]. Related to STAR Methods.

| | KBR [14] | LAC [14] | This work (blue) with cogen and FGC | D'Angelo et al. [15] SMR+CCS SG+FG scenario | Chisalita et al. [2] Case 1 | Wang et al. [16] | Green from [14] | This work (green) 60% efficiency | This work (green) 70% efficiency |
|---|---|---|---|---|---|---|---|---|---|
| $SC_{eq}$ [kWh/kg] | 7.92 | 7.69 | 13.48 | 16.46 | 10.55 | 11.75 | 8.61 | 10.90 | 9.47 |
| $\eta_{NH3eq}$ | 65.4% | 67.2% | 38.7% | 31.7% | 49.5% | 44.4% | 60.1% | 47.9% | 55.1% |

## Life Cycle Assessment

### Electricity Scenarios

For the conventional electricity scenarios, the current electricity mix for Saudi Arabia from the ecoinvent database [17] was used. For the renewable electricity scenarios (PV-based), the *570kWp open ground installation, multi-Si ecoinvent* activity was used and modified with the average solar irradiation for Saudi Arabia found in the ecoinvent activity *electricity production, photovoltaic, 3kWp slanted-roof installation, multi-Si, panel, mounted*. A wind electricity-based benchmark was included for green ammonia in **Section** *Climate Change Impacts based on*

*the Global Warming Potential (GWP100)*. **Table S7** summarizes the ecoinvent activities and modifications for the modelled electricity scenarios.

**Table S7.** Ecoinvent activities for each electricity scenario. Related to STAR Methods.

| **Electricity Scenario** | **Ecoinvent Activity** | **Location** | **Modifications** |
|---|---|---|---|
| Conventional | market for electricity, low voltage | SA | none |
| Renewable (PV-based) | electricity production, photovoltaic, 570kWp open ground installation, multi-Si | SA (adapted from RoW) | Technosphere inputs recalculated with Saudi Arabia average solar yield ($1499.6\ \frac{kWh}{kWp}$) |
| Wind (only for green ammonia) | electricity production, wind, 1-3MW turbine, onshore | RoW | none |

Background system processes that require electricity (e.g., tap water via desalination, nitrogen production) had their electricity technosphere flow replaced to match the modelled electricity scenario.

The process electricity requirements come from the compressors, pumps, and refrigeration. The refrigeration requirements ($Q$) are converted to an electricity requirement by assuming an ambient temperature of 50 °C for Saudi Arabia ($T_{ambient}$), a refrigerant temperature of -40 °C ($T_C$), and an exergetic efficiency of 0.6. The refrigeration power requirements are calculated via the equations below (*Equations 11-13*).

$$COP_{carnot} = \frac{T_C}{T_{ambient} - T_C} \qquad \text{(Equation 11)}$$

$$P_{ideal} = \frac{Q}{COP_{carnot}} \qquad \text{(Equation 12)}$$

$$P_{actual} = \frac{P_{ideal}}{exergetic\ efficiency} \qquad \text{(Equation 13)}$$

Hourly irradiation data for Saudi Arabia for the past five years from (JRC 2014) [18] was used to model the solar parks and corresponding battery systems.

To carry out the sensitivity on the PV panel efficiency, the activity "photovoltaic plant construction, 570kWp, multi-Si, on open ground" was modified and the parameter for PV efficiency ($\eta_{PV}$) was introduced. **Table S8** shows the modifications made to the activity.

**Table S8.** Modifications made to ecoinvent activity to model PV panel efficiency. Related to STAR Methods.

| Ecoinvent Activity | Technosphere flow | Amount | Equation Details |
|---|---|---|---|
| photovoltaic plant construction, 570kWp, multi-Si, on open ground | market for photovoltaic panel, multi-Si wafer | $\frac{570}{1\frac{kWp}{m^2} * \eta_{PV}} * (1 + loss_{panel})[m^2]$ | $loss_{panel} = 3\%$<br>$13.338\% \leq \eta_{PV} \leq 70\%$ |
| | market for photovoltaic mounting system, for 570kWp open ground module | $\frac{570}{1\frac{kWp}{m^2} * \eta_{PV}} [m^2]$ | |

## Green Ammonia

**Table S9** shows the inventories used for the LCA of green ammonia.

### *Green hydrogen*

The water requirement was calculated based on the stoichiometric amount needed per kg $H_2$ (9kg/kg H2), as done in [19]. Water was modelled using the ecoinvent process for desalinated water since the likely water source in Saudi Arabia is through desalination. The base electricity requirement was calculated based on an efficiency of 60% [19] which includes PEM electricity requirements in addition to water splitting. Hydrogen is produced at 30 bar and 60C.

**Table S9. [Green ammonia LCA inventory]** Inventories used to carry out LCA of green ammonia. Activities with replaced electricity to match scenario are indicated with a (*). Activities with their own inventories are indicated with a (**) and the inventory is included subsequently. Related to STAR Methods.

| Process | Material/Energy flow | Amount | Equation Details |
|---|---|---|---|
| **Green Ammonia Production (1kg ammonia)** | **Technosphere Flows** | | |
| | Nitrogen from Cryogenic Distillation** | $0.82kg$ | |
| | Hydrogen from PEM** | $0.18kg$ | |
| | Electricity* | $0.74kWh$ | |
| | Battery** (Ren Scenario) | $9.97\ x\ 10^{-5}\ kWh$ | |
| | Cooling Water** | $155.14kg$ | |
| | market for chemical factory, organics | $9.15\ x\ 10^{-11}\ unit$ | |
| **Nitrogen from Cryogenic Distillation (1kg nitrogen)** [15] | **Technosphere Flows** | | |
| | air separation facility construction | $4.43\ x\ 10^{-10}\ unit$ | |
| | Electricity* | $0.105kWh$ | |
| | Water, cooling, unspecified natural origin | $4\ x\ 10^{-3}\ m^3$ | |
| | **Biosphere Flows** | | |
| | Water | $1.5\ x\ 10^{-3}\ m^3$ | |
| **Cooling Water** [20] | **IHS process "COOLING WATER AT 75,000 GALLONS PER MINUTE"** | | |
| **Hydrogen from PEM (1kg hydrogen)** | **Technosphere Flows** | | |
| | tap water production, seawater reverse osmosis, conventional pretreatment, baseline module, single stage* | $9kg$ | |
| | Electrolyzer infrastructure ** | $1$ | |
| | Battery** (PV Scenario only, wind scenario does not consider battery) | $4.28\ x\ 10^{-3}\ kWh$ | |
| | Electricity* | $LHV_{H_2}/\eta_{elec}$ | $LHV_{H_2} = 33.33\ kWh/kg$ <br> $60\% \leq \eta_{elec} \leq 80\%$ |
| | heat production, natural gas, at boiler modulating <100kW | $0.18MJ$ [19] | |
| | **Biosphere Flows** | | |
| | Oxygen | $8kg$ | |
| **Electrolyzer infrastructure [19,21] (1kg $H_2$)** | **Technosphere Flows** | | |
| | Electrolyzer Stack** | $\frac{1}{elec_{out} \cdot \frac{1000 \cdot \eta_{elec}}{LHV_{H_2}} \cdot lt_{stack} \cdot 8760 \cdot f_{op}}$ | $elec_{out} = 1MW$ |
| | Electrolyzer Stack End of Life** | | $lt_{stack} = 7years$ [19] |
| | Electrolyzer Balance of Plant (BOP)** | $\frac{1}{elec_{out} \cdot \frac{1000 \cdot \eta_{elec}}{LHV_{H_2}} \cdot lt_{BOP} \cdot 8760 \cdot f_{op}}$ | $f_{op} = 34\%$ [19] |
| | Electrolyzer BOP End of Life** | | $lt_{BOP} = 20years$ [19] |
| **Electrolyzer Stack (1 MW capacity unit)** | **Technosphere Flows** | | |
| | activated carbon production, granular from hard coal | $9kg$ | |
| | aluminium production, primary, ingot | $27kg \cdot (1 - r_{aluminum})$ | $r_{aluminum} = 90\%$ |
| | copper production, cathode, solvent extraction and electrowinning process | $4.5kg \cdot (1 - r_{copper})$ | $r_{copper} = 70\%$ |
| | market for platinum | $0.075kg \cdot (1 - r_{platinum})$ | $r_{platinum} = 65\%$ |

| | | | |
|---|---|---|---|
| | market for platinum (platinum assumed as iridium from [21]) | $0.75kg \cdot (1 - r_{iridium})$ | $r_{iridium} = 25\%$ |
| | steel production, electric, chromium steel 18/8 | $100kg \cdot (1 - r_{steel})$ | $r_{steel} = 85\%$ |
| | tetrafluoroethylene production | $16kg$ | |
| | titanium production | $528kg \cdot (1 - r_{titanium})$ | $r_{titanium} = 91\%$ |
| **Electrolyzer Stack End of Life (1 MW capacity unit)** | **Technosphere Flows** | | |
| | Aluminum EOL** | $27kg$ | |
| | Copper EOL** | $4.5kg$ | |
| | Iridium EOL** | $0.75kg$ | |
| | Platinum EOL** | $0.075kg$ | |
| | Steel EOL** | $100kg$ | |
| | Titanium EOL** | $528kg$ | |
| | treatment of inert waste, inert material landfill | $25kg$ | |
| **Electrolyzer Balance of Plant (BOP) (1 MW capacity unit)** | **Technosphere Flows** | | |
| | aluminium production, primary, ingot | $100kg \cdot (1 - r_{aluminum})$ | |
| | copper production, cathode, solvent extraction and electrowinning process | $100kg \cdot (1 - r_{copper})$ | |
| | electronic component production, passive, unspecified | $1100kg$ | |
| | lubricating oil production | $200kg$ | |
| | polyethylene production, high density, granulate | $300kg$ | |
| | steel production, converter, low-alloyed | $4800kg \cdot (1 - r_{steel})$ | |
| | steel production, electric, chromium steel 18/8 | $1900kg \cdot (1 - r_{steel})$ | |
| | unreinforced concrete production, with cement CEM II/A | $2.33m^3$ | |
| **Electrolyzer BOP End of Life (1 MW capacity unit)** | **Technosphere Flows** | | |
| | Aluminum EOL** | $100kg$ | |
| | Copper EOL** | $100kg$ | |
| | Plastics EOL** | $1100kg$ | |
| | Polyethylene EOL** | $300kg$ | |
| | Steel EOL** | $6700kg$ | |
| | treatment of waste concrete, inert material landfill | $-5600kg$ | |
| | treatment of waste mineral oil, hazardous waste incineration | $-200kg$ | |
| **Plastics EOL (1 kg)** | **Technosphere Flows** | | |
| | treatment of waste plastic, mixture, sanitary landfill | $-0.025kg$ | |
| | treatment of waste plastic, mixture, municipal incineration | $-0.975kg$ | |
| **Polyethylene EOL (1 kg)** | **Technosphere Flows** | | |
| | market for waste polyethylene, for recycling, sorted | $0.277kg$ | |

| | | | |
|---|---|---|---|
| | treatment of waste polyethylene, municipal incineration | $-0.698kg$ | |
| | treatment of waste polyethylene, sanitary landfill | $-0.025kg$ | |
| **Aluminum EOL (1 kg)** | **Technosphere Flows** | | |
| | treatment of waste aluminium, sanitary landfill | $-(1 - r_{aluminum})$ | |
| **Copper EOL (1 kg)** | **Technosphere Flows** | | |
| | treatment of inert waste, inert material landfill | $-(1 - r_{copper}) \cdot 13\%$ | |
| | treatment of copper scrap by electrolytic refining | $-(1 - r_{copper}) \cdot 87\%$ | |
| **Iridium EOL (1 kg)** | **Technosphere Flows** | | |
| | treatment of inert waste, inert material landfill | $-(1 - r_{iridium})$ | |
| **Platinum EOL (1 kg)** | **Technosphere Flows** | | |
| | treatment of inert waste, inert material landfill | $-(1 - r_{platinum})$ | |
| **Steel EOL (1 kg)** | **Technosphere Flows** | | |
| | treatment of scrap steel, inert material landfill | $-(1 - r_{steel})$ | |
| **Titanium EOL (1 kg)** | **Technosphere Flows** | | |
| | treatment of inert waste, inert material landfill | $-(1 - r_{titanium})$ | |
| **Battery infrastructure (per kWh)** | **Inventory derived from Ellingsen et al.** [22] **More than 280 entries** | | |

*replaced electricity requirement with electricity scenario
**separate inventory provided

## Blue Ammonia

**Table S13** shows the inventories used for the LCA of blue ammonia (with and without flue gas capture (FGC)).

### *Natural gas supply chain*

The default ecoinvent processes for natural gas production and transportation for Algeria were used since this supply chain best resembles that of Saudi Arabia [23]. **Table S10** shows the ecoinvent activities and formulas used to model the natural gas supply chain per m$^3$ produced and transported. A parametrized variable $D_{NG}$ was introduced corresponding to the distance transported in km. The default transportation distance is 800km. The individual processes were modified as described below to implement the sensitivity analyses on the production and transportation leakage rates, where $\rho_{NG,AP} = 0.78\frac{kg}{m^3}$ corresponds to the natural gas density after processing [24].

**Table S10.** ecoinvent activities and formulas used to model the natural gas supply chain per m3 produced and transported. Related to STAR Methods.

| **Ecoinvent Activity** | **Location** | **Value** | **unit** |
|---|---|---|---|
| natural gas production | DZ | 1 | *m3* |
| transport, pipeline, onshore, long distance, natural gas | DZ | $\frac{\rho_{NG,AP}}{1000} * D_{NG}$ | *ton*km* |

The default ecoinvent NG production leakage for Algeria is 0.17% and corresponds to the emissions shown in **Table S11** per $m^3$ of natural gas produced. To carry out the sensitivity analysis, the emission components, $i$, were scaled with a parametrized leakage %, $L_{prod}$ according to Equation 14, where $\rho_{NG,BP}$ corresponds to the natural gas density before processing ($0.84 kg/m^3$) [24]. The default ecoinvent NG transportation leakage for Algeria is 0.2% per 1000km transported and corresponds to the emissions shown in **Table S12** per *ton km* of natural gas delivered.

$$emission_i(per\ m^3\ production) = \%\ of\ total_i * \rho_{NG,BP} * L_{prod} \quad (Equation\ 14)$$

**Table S11.** NG production leakage composition based on default Algerian production. Related to STAR Methods.

| **Emission Component ($i$)** | **Unit** | **% of total** |
|---|---|---|
| Methane | kg | 85.1% |
| Ethane | kg | 10.7% |
| non-methane volatile organic compounds (NMVOC) | kg | 1.7% |
| Propane | kg | 1.5% |
| Carbon Dioxide | kg | 0.8% |
| Butane | kg | 0.2% |

**Table S12.** NG transportation leakage composition based on default Algerian transportation. Related to STAR Methods.

| **Emission Component ($i$)** | **Unit** | **% of Total** |
|---|---|---|
| Methane | kg | 84.1% |
| Ethane | kg | 13.0% |
| Propane | kg | 2.6% |
| Butane | kg | 0.35% |

The ecoinvent functional unit for transportation is $1 ton\ km$. Therefore, for $1m^3$ of natural gas delivered, the distance to transport $1m^3$ of natural gas ($D_{unitized}$) must be calculated to unitize $1 ton\ km$. This is done via the following equation (*Equation 15*):

$$D_{unitized} = \frac{1}{\rho_{NG,AP}/1000} \quad (Equation\ 15)$$

To carry out the sensitivity analysis on the leakage rate per *ton km*, the emission components, $i$, were scaled with a parametrized leakage % per 1000 km, $L_{trans}$ according to Equation 16,

where $\rho_{NG,AP}$ corresponds to the natural gas density after processing ($0.78 kg/m^3$)[24]. The values are calculated per $m^3$ transported for $1 km$, and therefore $D_{unitized}$ is also included.

$$emission_i(per\ ton\ km\ transported) = \%\ of\ total_i * \rho_{NG,AP} * \frac{L_{trans}}{1000} * D_{unitized} \quad \text{(Equation 16)}$$

### *Carbon Capture and Storage (CCS)*

The post-capture operation consists of compression, transportation, and storage steps which each have an electricity requirement. The inventories are modelled according to [25,26]. The $CO_2$ coming out of the stripper is at 75 bar and needs to be compressed to 110 bar. The $CO_2$ is then transported to a storage facility, and the leakage per 1000km transported is also modelled based on [26] at 0.075% per 1000 km. It is *assumed* that the post-capture stream is homogeneous in all components throughout the transport and therefore the leakage rate is applied to all components in the post-capture stream. Only onshore transportation was considered.

### *Flue gas capture from cogeneration*

[27] and [28] provide the equations to calculate the MEA, power, and heat demand for the capture and storage of the flue gas from the cogeneration plant using an amine-based system. The system captures 69 ton$CO_2$/hour, 90% of the overall $CO_2$ emitted by the cogeneration plant. The capture system requires 85MW of heat and 10.3 MW of electricity without considering the electricity required to compress the captured $CO_2$ from 1 bar to 110bar. The main power requirements come from the blower for the flue gas to overcome the pressure drops in the scrubber column and the pump for MEA circulation between the scrubber and regeneration column. The system requires 1.5kg monoethanolamine (MEA) per ton $CO_2$ captured, and 2ppm MEA are released with the post-capture vent stream. These values were used to model the technosphere and biosphere flows of the flue gas capture system.

**Table S13. [Blue ammonia LCA inventory]** Inventories used to carry out LCA of blue ammonia. Some inventories are already included in **Table S9.** Activities with replaced electricity to match scenario are indicated with a (*). Activities with their own inventories are indicated with a (**) and the inventory is included subsequently. Related to STAR Methods.

| Process | Material/Energy flow | Amount | Equation Details |
|---|---|---|---|
| **Blue Ammonia Production, no FGC (1kg ammonia)** | **Technosphere Flows** | | |
| | Cogen System** | 1 | |
| | Natural Gas** | $1.2 m^3$ | |
| | market for methanol | $0.07 kg$ | |
| | Electricity* (not including cogen) | $1.7 kWh$ | |
| | heat production, natural gas, at boiler modulating <100kW (not including cogen) | $8.72 MJ$ | |
| | Battery** (Ren Scenario) | $1.28 \times 10^{-4}\ kWh$ | |
| | Cooling Water** | $308.65 kg$ | |
| | market for chemical factory, organics | $3.98 \times 10^{-10}\ unit$ | |
| | $CO_2$ Compression, Transport, and Storage** | $1.6 kg\ CO_2\ stored$ | |
| | tap water production, seawater reverse osmosis, conventional pretreatment, baseline module, single stage* | $1.76 kg$ | |
| | **Biosphere Flows** | | |
| | Carbon dioxide | $0.004 kg$ | |
| | Carbon monoxide | $4.43 \times 10^{-6}\ kg$ | |
| | Hydrogen | $8.53 \times 10^{-6}\ kg$ | |
| | Methane | $3.57 \times 10^{-5}\ kg$ | |
| | Methanol | $0.008 kg$ | |
| | Nitrogen | $5.23 \times 10^{-5}\ kg$ | |
| | Water | $9.3 \times 10^{-4}\ m^3$ | |
| **Cogen System (1kg ammonia)** | **Technosphere Flows** | | |
| | market for chemical factory, organics | $1.27 \times 10^{-10}\ unit$ | |
| | heat production, natural gas, at boiler modulating <100kW | $-10.93 MJ$ | |
| | Electricity* | $-0.4 kWh$ | |
| | **Biosphere Flows** | | |
| | Ammonia | $3.10 \times 10^{-11}\ kg$ | |
| | Carbon dioxide | $0.63 kg$ | |
| | Carbon monoxide | $3.17 \times 10^{-5}\ kg$ | |
| | Hydrogen | $2.74 \times 10^{-6}\ kg$ | |
| | Methane | $3.58 \times 10^{-24}\ kg$ | |
| | Methanol | $6.20 \times 10^{-23}\ kg$ | |
| | Nitrogen | $4.7 kg$ | |
| | Oxygen | $0.065 kg$ | |
| | Water | $9.5 \times 10^{-4}\ m^3$ | |
| **Natural Gas (1m³)** *(details found in Natural Gas section)* | **Technosphere Flows** | | |
| | natural gas production | $1\ m^3$ | |
| | transport, pipeline, onshore, long distance, natural gas | $\frac{\rho_{NG,AP}}{1000} * D_{NG}[ton * km]$ | $\rho_{NG,AP} = 0.78 \frac{kg}{m^3}$ 24<br>$0km \leq D_{NG} \leq 1500km$ |
| **$CO_2$ Compression, Transport, and** | **Technosphere Flows** | | |
| | $CO_2$ Compression** | $1 kg$ | |

| | | | |
|---|---|---|---|
| **Storage (1kg stored)** [25,26] | $CO_2$ Transport** | $\frac{1}{1000} * D_{CO_2}[ton * km]$ | $0km \leq D_{CO_2} \leq 1500km$ |
| | $CO_2$ Storage** | $1kg$ | |
| **$CO_2$ Compression (1 kg $CO_2$ at 11MPa)** | **Technosphere Flows** | | |
| | Electricity* | $0.0094kWh$ | |
| | $CO_2$ Compressor Construction** | $1.61\ x\ 10^{-11}\ unit$ | |
| | **Biosphere Flows** (0.029% leakage) | | |
| | Ammonia | $0kg$ | |
| | Carbon dioxide | $2.62\ x\ 10^{-4}\ kg$ | |
| | Carbon monoxide | $1.15\ x\ 10^{-6}\ kg$ | |
| | Hydrogen | $6.24\ x\ 10^{-7}\ kg$ | |
| | Methane | $4.62\ x\ 10^{-6}\ kg$ | |
| | Methanol | $1.15\ x\ 10^{-5}\ kg$ | |
| | Nitrogen | $9.98\ x\ 10^{-6}\ kg$ | |
| | Oxygen | $0kg$ | |
| | Water | $6.77\ x\ 10^{-7}\ kg$ | |
| **$CO_2$ Compressor Construction (1 unit)** | **Technosphere Flows** | | |
| | market for concrete, normal | $65\ m^3$ | |
| | market for copper, anode | $7000kg$ | |
| | market for steel, chromium steel 18/8, hot rolled | $3250kg$ | |
| | market for reinforcing steel | $61750kg$ | |
| | market for diesel, burned in building machine | $1.98\ x\ 10^6 MJ$ | |
| | polyethylene production, low density, granulate | $20000kg$ | |
| | market for scrap copper | $7000kg$ | |
| | market for waste reinforcement steel | $65000kg$ | |
| | market for waste polyethylene | $20000kg$ | |
| | market for waste concrete | $1.55\ x\ 10^5 kg$ | |
| | Electricity* | $61000kWh$ | |
| **$CO_2$ Transport (1 ton*km)** | **Technosphere Flows** | | |
| | Electricity* | $0.012kWh$ | |
| | $CO_2$ Onshore Pipeline Construction** | $4.27\ x\ 10^{-6}\ [\frac{ton \cdot km}{hr}\ capacity]$ | Lifetime of 30 years, 7800hrs/year |
| | **Biosphere Flows** (0.075% per 1000 km) | | |
| | Ammonia | $0kg$ | |
| | Carbon dioxide | $6.71\ x\ 10^{-4}\ kg$ | |
| | Carbon monoxide | $2.94\ x\ 10^{-6}\ kg$ | |
| | Hydrogen | $1.6\ x\ 10^{-6}\ kg$ | |
| | Methane | $1.18\ x\ 10^{-5}\ kg$ | |
| | Methanol | $2.96\ x\ 10^{-5}\ kg$ | |
| | Nitrogen | $2.56\ x\ 10^{-5}\ kg$ | |
| | Oxygen | $0kg$ | |
| | Water | $1.74\ x\ 10^{-6}\ kg$ | |
| **$CO_2$ Onshore Pipeline Construction (1 ton*km capacity)** | **Technosphere Flows** | | |
| | drawing of pipe, steel | $597.45kg$ | |
| | market for sand | $4854kg$ | |
| | diesel, burned in building machine | $3261MJ$ | |
| | market for transport, freight train | $125.3ton * km$ | |
| | market for transport, freight, lorry >32 metric ton, EURO6 | $64.2ton * km$ | |

| | | | |
|---|---|---|---|
| | reinforcing steel production | $597.45kg$ | |
| | bitumen seal production | $5.77kg$ | |
| | polyethylene production, low density, granulate | $11.55kg$ | |
| | market for waste polyethylene | $5.77kg$ | |
| | market for waste bitumen | $2.89kg$ | |
| | market for inert waste | $2427.13kg$ | |
| | market for waste reinforcement steel | $298.7kg$ | |
| | gas turbine construction, 10MW electrical | $1.4 \, x \, 10^{-5} \, unit$ | |
| **$CO_2$ Storage (1 kg $CO_2$)** | **Technosphere Flows** | | |
| | Electricity* | $0.007kWh$ | |
| | $CO_2$ Storage Construction** | $3.8 \, x \, 10^{-9} \, [\frac{ton}{hr} capacity]$ | |
| | **Biosphere Flows** | | |
| | Water | $6.03 \, x \, 10^{-8} \, m^3$ | |
| **$CO_2$ Storage Construction (1 ton/hr capacity)** | **Technosphere Flows** | | |
| | offshore well production, oil/gas | $4.44m$ | |
| | offshore platform production, natural gas | $0.0043unit$ | |
| **Blue Ammonia Production, FGC (1kg ammonia)** | **Technosphere Flows** | | |
| | Blue Ammonia Production, no FGC** | 1 | |
| | Flue Gas Capture** | 1 | |
| **Flue Gas Capture (1kg ammonia)** | **Technosphere Flows** | | |
| | market for monoethanolamine | $8.4 \, x \, 10^{-4} kg$ | |
| | market for chemical factory, organics | $1.27 \, x \, 10^{-11} \, unit$ | |
| | heat production, natural gas, at boiler modulating <100kW | $2.5MJ$ | |
| | Electricity* | $0.08kWh$ | |
| | $CO_2$ Compression, Transport, and Storage (FGC)** | $0.56kg \, CO_2 \, stored$ | |
| | Cooling Water** | $60kg$ | |
| | **Biosphere Flows** | | |
| | Carbon dioxide | $-0.56kg$ | |
| | Monoethanolamine | $1.16 \, x \, 10^{-5} kg$ | |
| **$CO_2$ Compression, Transport, and Storage (FGC) (1kg stored)** [25,26] | **Technosphere Flows** | | |
| | $CO_2$Compression FGC** | $1kg$ | |
| | $CO_2$ Transport FGC** | $\frac{1}{1000} * D_{CO_2} [ton * km]$ | $0km \leq D_{CO_2} \leq 1500km$ |
| | $CO_2$ Storage** | $1kg$ | |
| **$CO_2$ Compression FGC (1 kg CO2 at 11MPa)** | **Technosphere Flows** | | |
| | Electricity* | $0.126kWh$ | |
| | $CO_2$ Compressor Construction** | $1.61 \, x \, 10^{-11} \, unit$ | |
| | **Biosphere Flows** (0.029% leakage) | | |
| | Carbon dioxide | $2.9 \, x \, 10^{-4} \, kg$ | |
| **$CO_2$ Transport FGC (1 ton*km)** | **Technosphere Flows** | | |
| | Electricity* | $0.012kWh$ | |
| | $CO_2$Onshore Pipeline Construction** | $4.27 \, x \, 10^{-6} \, [\frac{ton*km}{hr} capacity]$ | Lifetime of 30 years, 7800hrs/year |
| | **Biosphere Flows** (0.075% per 1000 km) | | |
| | Carbon dioxide | $7.4 \, x \, 10^{-4} \, kg$ | |

*replaced electricity requirement with electricity scenario
**separate inventory provided

## REFERENCES


1. Smith, C., Hill, A.K., and Torrente-Murciano, L. (2020). Current and future role of Haber–Bosch ammonia in a carbon-free energy landscape. Energy & Environmental Science *13*, 331–344-331–344.
2. Chisalita, D.A., Petrescu, L., and Cormos, C.C. (2020). Environmental evaluation of european ammonia production considering various hydrogen supply chains. Renewable & Sustainable Energy Reviews *130*. ARTN 109964
10.1016/j.rser.2020.109964.
3. House, K.Z., Baclig, A.C., Ranjan, M., Van Nierop, E.A., Wilcox, J., and Herzog, H.J. (2011). Economic and energetic analysis of capturing CO2 from ambient air. Proceedings of the National Academy of Sciences *108*, 20428-20433.
4. Wilcox, J., Psarras, P.C., and Liguori, S. (2017). Assessment of reasonable opportunities for direct air capture. Environmental Research Letters *12*, 065001.
5. Gomez, J.R., Baca, J., and Garzon, F. (2020). Techno-economic analysis and life cycle assessment for electrochemical ammonia production using proton conducting membrane. International Journal of Hydrogen Energy *45*, 721-737. 10.1016/j.ijhydene.2019.10.174.
6. Bicer, Y., and Dincer, I. (2017). Life cycle assessment of nuclear-based hydrogen and ammonia production options: A comparative evaluation. International Journal of Hydrogen Energy *42*, 21559–21570-21559–21570.
7. Liu, X.Y., Elgowainy, A., and Wang, M. (2020). Life cycle energy use and greenhouse gas emissions of ammonia production from renewable resources and industrial by-products. Green Chemistry *22*, 5751-5761. 10.1039/d0gc02301a.
8. Mohamed, A.M.O., Al-Ghamdi, S.G., and Bicer, Y. (2021). Life cycle assessment of clean ammonia synthesis from thermo-catalytic solar cracking of liquefied natural gas. International Journal of Hydrogen Energy *46*, 38551–38562-38551–38562.
9. Makhlouf, A., Serradj, T., and Cheniti, H. (2015). Life cycle impact assessment of ammonia production in Algeria: A comparison with previous studies. Environmental Impact Assessment Review *50*, 35–41-35–41.
10. Armijo, J., and Philibert, C. (2020). Flexible production of green hydrogen and ammonia from variable solar and wind energy: Case study of Chile and Argentina. International Journal of Hydrogen Energy *45*, 1541-1558. 10.1016/j.ijhydene.2019.11.028.
11. Tuna, P., Hulteberg, C., and Ahlgren, S. (2014). Techno-Economic Assessment of Nonfossil Ammonia Production. Environmental Progress & Sustainable Energy *33*, 1290-1297. 10.1002/ep.11886.
12. Zhang, H.F., Wang, L.G., Van Herle, J., Marechal, F., and Desideri, U. (2020). Techno-economic comparison of green ammonia production processes. Applied Energy *259*. ARTN 114135
10.1016/j.apenergy.2019.114135.
13. Nosherwani, S.A., and Neto, R.C. (2021). Techno-economic assessment of commercial ammonia synthesis methods in coastal areas of Germany. Journal of Energy Storage *34*. ARTN 102201
10.1016/j.est.2020.102201.
14. del Pozo, C.A., and Cloete, S. (2022). Techno-economic assessment of blue and green ammonia as energy carriers in a low-carbon future. Energy Conversion and Management *255*. ARTN 115312
10.1016/j.enconman.2022.115312.

15. D'Angelo, S.C., Cobo, S., Tulus, V., Nabera, A., Martín, A.J., Pérez-Ramírez, J., and Guillén-Gosálbez, G. (2021). Planetary Boundaries Analysis of Low-Carbon Ammonia Production Routes. ACS Sustainable Chemistry & Engineering *9*, 9740–9749-9740–9749.
16. Wang, M., Khan, M.A., Mohsin, I., Wicks, J., Ip, A.H., Sumon, K.Z., Dinh, C.T., Sargent, E.H., Gates, I.D., and Kibria, M.G. (2021). Can sustainable ammonia synthesis pathways compete with fossil-fuel based Haber-Bosch processes? Energy & Environmental Science *14*, 2535-2548. 10.1039/d0ee03808c.
17. Wernet, G., Bauer, C., Steubing, B., Reinhard, J., Moreno-Ruiz, E., Weidema, B. (2016). The ecoinvent database version 3 (part I): overview and methodology. The International Journal of Life Cycle Assessment. Available at: http://link.springer.com/10.1007/s11367-016-1087-8.
18. European Commission Joint Research Centre, I.f.E.a.T.I. (2014). Solar radiation and photovoltaic electricity potential country and regional maps.
19. Bareiß, K., de La Rua, C., Möckl, M., and Hamacher, T. (2019). Life cycle assessment of hydrogen from proton exchange membrane water electrolysis in future energy systems. Applied Energy *237*, 862–872-862–872.
20. IHS (2017). Process economic yearbook.
21. Baumgärtner, N., Deutz, S., Reinert, C., Nolzen, N., Kuepper, L.E., Hennen, M., Hollermann, D.E., and Bardow, A. (2021). Life-Cycle Assessment of Sector-Coupled National Energy Systems: Environmental Impacts of Electricity, Heat, and Transportation in Germany Till 2050. Frontiers in Energy Research *9*. 10.3389/fenrg.2021.621502.
22. Ellingsen, L.A.-W., Majeau-Bettez, G., Singh, B., Srivastava, A.K., Valøen, L.O., and Strømman, A.H. (2014). Life Cycle Assessment of a Lithium-Ion Battery Vehicle Pack. Journal of Industrial Ecology *18*, 113–124-113–124.
23. Saudi Aramco Sustainability Report 2021. (2021). Saudi Aramco, 104.
24. Schori, S., and Frischknecht, R. (2012). Life Cycle Inventory of Natural Gas Supply.
25. Shu, D.Y., Deutz, S., Winter, B.A., Baumgärtner, N., Leenders, L., and Bardow, A. (2023). The role of carbon capture and storage to achieve net-zero energy systems: Trade-offs between economics and the environment. Renewable and Sustainable Energy Reviews *178*, 113246. https://doi.org/10.1016/j.rser.2023.113246.
26. Koornneef, J., van Keulen, T., Faaij, A., and Turkenburg, W. (2008). Life cycle assessment of a pulverized coal power plant with post-combustion capture, transport and storage of CO2. International Journal of Greenhouse Gas Control *2*, 448–467-448–467.
27. Abu-Zahra, M.R.M., Schneiders, L.H.J., Niederer, J.P.M., Feron, P.H.M., and Versteeg, G.F. (2007). CO2 capture from power plants. International Journal of Greenhouse Gas Control *1*, 37–46-37–46.
28. Geuzebroek, F.H., Schneiders, L.H.J.M., Kraaijveld, G.J.C., and Feron, P.H.M. (2004). Exergy analysis of alkanolamine-based CO2 removal unit with AspenPlus. Energy *29*, 1241–1248-1241–1248.
